# Concept-Aware Learning Framework for Detecting Common Symptoms in Social Media Text: Model Development and Evaluation Study

**DOI:** 10.2196/94769

**Published:** 2026-10-15

**Authors:** Michael Van Supranes, Shaowen Peng, Tomohiro Nishiyama, Shoko Wakamiya, Eiji Aramaki

**Affiliations:** 1 Nara Institute of Science and Technology Ikoma, Nara Japan; 2 University of the Philippines Diliman Quezon City, Metro Manila Philippines

**Keywords:** natural language processing, public health surveillance, symptom detection, model robustness, data augmentation, large language model, medical NLP, medical natural language processing

## Abstract

**Background:**

Social media offers valuable information for public health surveillance, but detecting valid symptom expressions in noisy and informal text remains challenging. Fine-tuned language models can achieve strong test accuracy while relying on spurious patterns, limiting their reliability in real-world applications.

**Objective:**

This study aimed to develop a more robust symptom detection model by incorporating surveillance-relevant attributes into the training objective and introducing a counterfactual-based evaluation framework to assess attribute sensitivity.

**Methods:**

We proposed a concept-aware learning framework (CALF), which combines multiattribute counterfactual data augmentation with multitask learning. We compared standard fine-tuning (baseline) with data-level interventions, a model-level intervention, hybrid strategies, and prompt-based methods across multiple encoders and LLMs for data augmentation. Performance was evaluated on the following 3 datasets: the MedWeb test using macro *F*_1_-score (m-*F*_1_), the MedWeb checklist using flip accuracy (FA), and a supplementary test set using *F*_1_-score for flu-or-fever detection (f-*F*_1_). FA measures sensitivity to surveillance-relevant attributes, and the supplementary test score serves as an indicator for out-of-distribution performance.

**Results:**

The baseline achieved an m-*F*_1_-score of 0.871 on the test set but a low overall FA on the checklist (FA=0.342). It also has limited sensitivity to recovery status, timing of occurrence, and uncertainty of expression. Compared with the baseline, CALF has a comparable MedWeb test score (m-*F*_1_=0.880 vs 0.871; *P*=.18), but a substantially higher FA (FA=0.830 vs 0.342; *P*=.001) and supplementary test score (f-*F*_1_=0.856 vs 0.797; *P*=.007). CALF also improved sensitivity to recovery, timing, and uncertainty across pretrained encoders. The gains were generally larger when GPT-4.1 (OpenAI) was used for data augmentation. Under GPT-4.1, CALF often achieved the highest overall FA among encoder-based methods. When Qwen2.5-7B-Instruct (Alibaba Cloud) was used, an alternative hybrid strategy may achieve comparable or slightly better results. Checklist performance was strongly associated with supplementary test performance (ρ=0.811), whereas MedWeb test performance showed weak association with supplementary test performance (ρ=–0.042). Prompting GPT-4.1 achieved the highest FA overall (FA=0.920) and the highest supplementary test score (f-*F*_1_=0.910), but a lower test set score than the baseline (m-*F*_1_=0.776 vs 0.871).

**Conclusions:**

Standard held-out metrics alone do not adequately characterize robustness to dataset-specific or spuriously correlated features. Models may perform well on the test set while remaining insensitive to attributes that influence whether a symptom mention is relevant to surveillance. The MedWeb checklist complements conventional evaluation by measuring sensitivity to controlled attribute changes and may indicate out-of-distribution performance. CALF improved sensitivity to surveillance-relevant attributes while maintaining strong classification performance, supporting concept-aware learning as a practical strategy for developing more robust encoder-based models.

## Introduction

### Background

The rapid growth of user-generated content on social media has created new opportunities for public health surveillance. Social media data offer a large and continuously updated stream of text that can support early detection of disease outbreaks and population-level symptom trends [1-5]. However, realizing this potential requires models that can reliably identify valid symptom reports in noisy and informal text. Unlike clinical records, symptom expressions on social media are very dynamic and context-dependent. A symptom may be implied rather than explicitly stated. For example, the post “I will have cold medicine and go to bed now” suggests the presence of a cold. In contrast, some posts mention symptom terms without indicating an actual case. The sentence “I hope I’m not gonna get a cold” includes the word “cold,” but it is purely speculative. Similarly, symptoms may be described retrospectively (“Last month, I had a fever”), hypothetically (“If I go out, I might get flu.”), or as fully resolved (“My congestion has been long gone”). These variations illustrate that mere presence of a symptom-related word does not necessarily imply an active or self-reported case. Misclassifying such posts may lead to inflated symptom prevalence estimates and misleading surveillance signals.

Recent works on symptom detection have increasingly adopted state-of-the-art natural language processing (NLP) techniques, including fine-tuned transformer-based models and, more recently, large language models (LLMs) [5-12]. These approaches have led to substantial gains in symptom detection and extraction accuracy. However, empirical evidence shows that LLM performance varies notably across data sources, target symptoms, and languages [9,10,12], and that supervised fine-tuning is prone to learning data-specific biases [13-15]. Despite this, to our knowledge, most symptom detection studies have given limited attention to model robustness, particularly with respect to confounding textual features and generalization beyond the training distribution. Moreover, while cross-dataset, adversarial, or counterfactual-based evaluations have been developed in other NLP applications [16-20], recent symptom detection studies often rely on a single standard test split for evaluation. These limitations underscore a gap in both the development and evaluation of robust symptom detection models.

One reason for this limitation is that standard supervised training does not explicitly guide models to focus on surveillance-relevant concepts. This allows models to exploit “shortcuts” or spurious correlations between textual features and target labels. For example, a model trained to detect recent, active symptom cases may simply associate symptom-related terms with positive labels without accounting for the background context. To address this limitation, we propose a concept-aware learning framework (CALF) that allows one to explicitly incorporate surveillance-relevant concepts into the learning objective. It combines targeted data augmentation with multitask learning. In addition, we design a counterfactual-based test for evaluating model robustness. Figure 1 presents an overview of the proposed approach and the counterfactual-based test.

**Figure 1:**
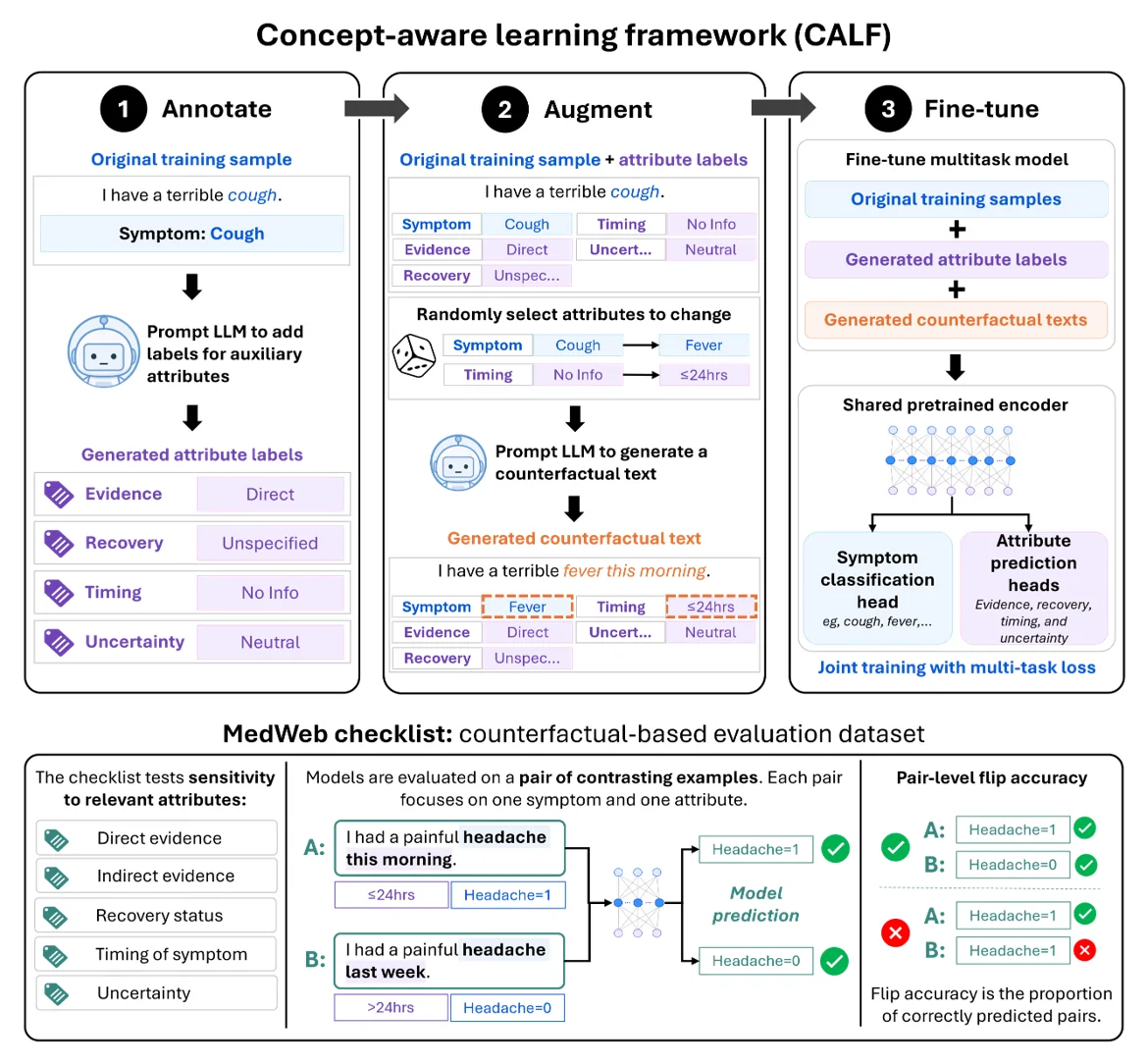
Overview of the concept-aware learning framework (CALF) and MedWeb Checklist evaluation. CALF uses researcher-defined attributes that are relevant to symptom surveillance. First, a large language model (LLM) annotates each training text with attribute labels, such as evidence, recovery, timing, and uncertainty. Second, the LLM generates counterfactual texts by modifying the symptom label and selected attributes, such as changing the timing of a symptom from unspecified to within 24 hours. Third, a pretrained language model is fine-tuned using a multitask objective that jointly predicts symptoms and attribute labels. The MedWeb Checklist evaluates robustness using minimally contrasting text pairs that differ in one attribute. A pair is counted as correct only when the model predicts the correct symptom labels for both texts, as measured by flip accuracy (FA).

### Related Work

Model robustness is influenced by both the training data and optimization strategy. Accordingly, existing approaches to improving robustness broadly fall into data-level interventions, model-level interventions, or combinations of the 2. In medical NLP, both data-level and model-level methods have been explored to improve robustness [21-24]. However, these approaches are rarely the focus in studies for symptom detection and surveillance. The closest related work is limited to health mention classification [25].

Data-level interventions improve robustness by modifying or augmenting training data. Synthetic data generation has been shown to improve generalization across NLP tasks [26-28], but may also amplify biases or introduce unintended artifacts [29,30]. Counterfactual data augmentation (CDA) addresses this limitation by introducing minimally modified examples that help weaken spurious correlations or emphasize task-relevant features [31-34]. CDA methods differ in how counterfactuals are constructed. Some approaches modify token-level or sentence-level features associated with spurious correlations [35-37], but they often rely on cross-dataset comparisons. It may also fail to capture higher-level, concept-based confounding features [38]. Other approaches focus on predefined attributes, which may be more feasible in domains such as symptom detection where surveillance criteria are known. Previous works suggest that modifying multiple relevant attributes can yield stronger robustness gains [39,40]. CALF adopts this multiattribute CDA paradigm but extends it beyond data augmentation.

Model-level interventions enhance robustness by modifying training objectives or model architecture. Standard supervised fine-tuning provides no explicit guidance on which features should drive predictions, allowing reliance on spurious correlations. To address this limitation, multitask learning has been used to jointly train models on related tasks or attributes [41-45]. However, multitask learning requires additional annotations, increasing data collection costs. Recent work has explored hybrid approaches combining multitask learning with data augmentation, showing promise across NLP tasks [36,46]. CALF follows this hybrid paradigm but differs from previous approaches by operating on predefined, concept-level attributes rather than token-level perturbations, paraphrastic variation, or cross-dataset methods.

### Goals of the Study

This study aims to investigate and improve the robustness of symptom detection models applied to social media text through a CALF. Specifically, we evaluate the following hypotheses:

- Strong classification accuracy on the test set does not guarantee sensitivity to surveillance-relevant features. Hence, it does not necessarily imply robustness to data-specific or spuriously correlated features.
- CALF helps improve robustness to spurious features by increasing the fine-tuned model’s sensitivity to surveillance-relevant attributes. Specifically, this study covers directness of evidence, timing of occurrence, recovery status, and uncertainty of expression.
- Models trained using the CALF have better overall robustness to data-specific or spuriously correlated features than models trained using standard supervised fine-tuning, data-augmentation-only, multi-task-learning-only, and alternative hybrid methods.

## Methods

### Overview

We conducted controlled experiments on the MedWeb task dataset [47] to evaluate the research hypotheses. To assess robustness, we introduced the MedWeb checklist dataset, which contains minimally contrasting examples or counterfactuals that systematically vary surveillance-relevant attributes. In addition, we constructed a small supplementary test set using 200 cases from the SPEED++ [48] and MedNLP-SC [49] datasets. This supplementary evaluation was used to examine whether the observed findings could extend beyond the MedWeb dataset.

The primary experiments compared the proposed method, CALF, with data-level interventions, model-level interventions, and alternative hybrid approaches. Methods requiring synthetic data generation or LLM-based annotation use GPT-4.1 (OpenAI). When synthetic data generation is required, 2000 additional training samples were generated using the specified data augmentation method, 1000 true cases and 1000 noncases. Unless otherwise stated, all models are trained by fine-tuning a RoBERTa (Robustly optimized BERT approach)-large model [50]. We additionally evaluated 2 prompting baselines to compare the encoder-based approaches with direct LLM-based classification. These baselines used an instruction-based prompt adapted from previous work on the MedWeb dataset [12], and they did not involve model fine-tuning. GPT-4.1 and Qwen2.5-7B-Instruct (Alibaba Cloud) were evaluated for this purpose.

To assess consistency across pretrained representations, selected methods are additionally evaluated using BERTweet, ClinicalBERT, and BioMed-RoBERTa. We also repeated the selected data augmentation experiments using Qwen2.5-7b-Instruct in place of GPT-4.1. Finally, we performed a qualitative error analysis.

### Materials

#### MedWeb Task Dataset

The MedWeb task dataset [47] serves as the primary dataset in this study. It consists of short, tweet-like messages annotated for a multilabel symptom classification. Each instance is labeled with binary indicators for the following 8 symptoms or diseases: cold, cough, diarrhea, influenza, fever, hay fever, headache, and runny nose. Following the original annotation guidelines [51], a symptom is labeled positive only if it is described as occurring within 24 hours and within the speaker’s vicinity, reflecting the dataset’s intended use for real-time symptom surveillance. The training split contains 1920 instances, and the test split contains 640 instances. Fever and runny nose are the most frequently observed symptoms, while hay fever and influenza are the least represented cases in the data. The distribution of the training and test sets by symptom or disease is in Multimedia Appendix 1.

#### MedWeb Checklist Dataset

To complement the standard test set, we constructed the *MedWeb checklist dataset*, an evaluation dataset designed to assess models’ robustness in terms of sensitivity to surveillance-relevant attributes. In total, four attributes were considered: (1) type of evidence, (2) timing of occurrence, (3) recovery status, and (4) uncertainty of expression*.* Refer to Table 1 for description and examples.

These attributes were selected because they influence symptom-labeling decisions in common scenarios described in the MedWeb annotation guidelines and are routinely considered when determining whether a social media post reflects a valid, surveillance-relevant symptom mention. They can also be inferred from the text alone, and based on the validation results, they can be controlled reasonably well during counterfactual generation. Other factors, such as location, symptom severity, or nonliteral uses of symptom-related terms, may also affect model behavior. Location was excluded because it may require post metadata or assumptions about the user’s location. Nonliteral usage was also excluded because it cannot be applied consistently across symptoms; for example, “cold” may refer to temperature rather than illness, whereas other symptom terms do not have similarly natural alternative meanings. Severity is reflected indirectly in some evidence expressions, such as a high body temperature, a nose “leaking like a faucet,” or “nonstop visits to the toilet,” but it was not explicitly controlled as a separate attribute. The current checklist should therefore be viewed as a focused and parsimonious evaluation rather than a comprehensive taxonomy. Future studies may extend the framework to additional attributes, provided that they are clearly defined, relevant to the task, and can be reliably generated, annotated, and validated.

**Table 1:** Description of surveillance-relevant attributes in the MedWeb checklist data.

Attribute	Description	Levels and examples
Evidence	Indicates whether the symptom is expressed directly, or indirectly through context. This does not affect the label, but it affects complexity of detection.	Direct: “I have a cough.”, clear positive symptom. Indirect: “The doctor gave me cough syrup.”, symptom is implied; still labeled positive.
Timing	Refers to when the symptom occurred relative to the posting time. Surveillance is only concerned with recent or active cases.	Within 24 hours (recent or active): “My runny nose started this morning.”, labeled positive. More than 24 hours ago (past or inactive): “I had a fever last week.”, labeled negative. Unspecified: No explicit details about the timing of occurrence.
Recovery	Indicates whether the person with a symptom has recovered and to what extent.	Full Recovery: “I had cough, but I’m fully recovered now.”, labeled negative. Partial or ongoing: “Feeling better, but the cough is still here.”, labeled positive. No info: Recovery is not mentioned.
Uncertainty	Refers to uncertain expressions about symptoms. Hypothetical statements are labeled negative, while doubtful or questioning expressions are labeled positive because they indicate a possible symptom.	Hypothetical or conditional: “If I stay up late, I’ll get a fever.”, labeled negative. Doubtful or questioning: “I think I have fever. Is this real?”, labeled positive since symptom is a possibility. Normal or neutral: Symptom is not expressed with uncertainty.

The checklist dataset is inspired by the concept-level causal effect framework [52], in which the effect of a specific attribute is assessed by comparing model predictions across minimally contrasting examples that differ only with respect to those attributes. For example, consider the statements: “I went on a trip and got a sunburn as a souvenir.” and “I went on a trip and brought back a headache as a souvenir.” These sentences are identical except for the symptom mentioned (“sunburn” vs “headache”). Because all other aspects of the text are held constant, any change in model prediction can be attributed to the difference in the symptoms displayed. This design enables isolation of the influence of each attribute on model behavior. Accordingly, robustness is defined in this study as a model’s ability to produce predictions that are consistent with the underlying attribute structure of the text, rather than relying on spurious or dataset-specific cues.

A total of 337 counterfactual sets were included in the final dataset, with at least 35 sets constructed for each symptom category. Each set consists of 9 counterfactual variants from a shared base text. This enables evaluation of sensitivity to direct evidence (explicit mention vs no evidence), indirect evidence (implied case vs no evidence), recent occurrence (within 24 hours vs outside 24 hours), recovery status (fully recovered vs partially recovered), and uncertainty of expression (speculative only vs doubtful). In total, the checklist dataset contains 3033 texts. The distribution of samples in the checklist data and examples of counterfactual sets are included in Multimedia Appendix 1.

The checklist dataset was constructed by using test cases as seed texts for counterfactual text generation. To reduce the risk of circular evaluation, the checklist dataset was intentionally designed to differ from the training data augmentation process in several key aspects: data source (held-out test set vs training set), LLM model version (OpenAI’s GPT-5 vs GPT-4.1), prompt strategy (stage-wise attribute-specific prompts vs single-pass generation), counterfactual generation (1 attribute at a time vs multiple attributes simultaneously), and quality control (LLM-as-a-judge or editor vs none). These differences reflect the distinct purposes of the 2 pipelines. Training augmentation represents a practical setting in which multiple attributes may interact naturally within a post. It also prioritizes efficiency, as synthetic-data generation should remain simpler and less costly than collecting and manually annotating additional data. In contrast, checklist construction isolates one attribute at a time to assess model sensitivity to controlled changes more directly.

#### Manual Validation of Counterfactual Text Generation

The generated counterfactual sets underwent a multistage quality-assurance procedure. First, GPT-5 evaluated each generated text using a 7-point Likert scale for attribute alignment, linguistic naturalness, and semantic consistency. Only sets receiving a score of at least 5 on every dimension for all variants were retained, resulting in an initial pool of 387 sets. Second, sampled counterfactual texts were independently reviewed by 2 graduate-level researchers with NLP backgrounds. Both raters identified the intended attribute in 73 of 100 (73%) sampled texts for type of evidence, 98 of 100 (98%) for recovery status, 95 of 100 (95%) for timing of occurrence, and 74 of 100 (74%) for uncertainty of expression. Disagreements occurred mainly for indirect symptom expressions and uncertain statements; nevertheless, at least 1 rater identified the intended attribute in more than 90 of the 100 evaluated texts for each attribute. Finally, the researchers reviewed the rater comments and manually examined the remaining candidate sets, removing texts that primarily expressed a question, described a hypothetical or pretended symptom, or referred to a nonhuman subject. Examples include “How do you say hay fever in English?,” “I pretend to have influenza,” and “I wonder if dogs get things like a stuffy nose?.” This final review reduced the dataset from 387 to 337 sets. Full details of the construction and quality-assurance procedures are provided in Multimedia Appendix 2.

#### Supplementary Test Set

To explore potential out-of-distribution performance beyond MedWeb, we constructed a small curated supplementary test set using posts from SPEED++ [48] and MedNLP-SC [49]. The selected posts were manually inspected and relabeled using the MedWeb annotation guidelines. Given the limited number of manually labeled examples, this analysis was intended as an exploratory external evaluation rather than a comprehensive validation study.

SPEED++ primarily contains COVID-19–related posts, many of which are delayed self-reports, general informational statements, or posts without explicit MedWeb-relevant symptoms. To increase the number of informative positive examples, selected borderline cases were minimally edited to clarify symptom timing, recovery status, or the presence of relevant symptoms while preserving the original language and context. For example, the post “I’ve been sick for 2 weeks and I’m survived! Coronavirus isn’t soooo bad” was revised to “I’ve been sick for 2 weeks, down with a fever, but I survived! Getting better. Coronavirus isn’t soooo bad.” Additional examples are provided in Multimedia Appendix 2. MedNLP-SC was originally developed for adverse drug reaction detection and includes posts mentioning symptoms such as fever and fatigue. Selected MedNLP-SC posts were therefore relabeled according to the MedWeb annotation guidelines, focusing on the symptoms expressed in the text rather than their attribution to an adverse drug reaction.

The final set included 35 positive and 65 negative SPEED++ posts, and 49 positive and 51 negative MedNLP-SC posts. A prediction was considered correct when the model identified either influenza or fever for a positive case and neither symptom for a negative case. This combined criterion was used because influenza-positive examples in MedWeb consistently co-occur with a positive fever label and because some COVID-19–related and adverse-reaction posts describe flu-like symptom combinations.

### CALF

#### CALF Overview

CALF is the proposed fine-tuning strategy in this study (Figure 1). It is a hybrid approach that integrates multiattribute CDA with multitask learning to explicitly promote sensitivity to surveillance-relevant concepts.

CALF assumes that a set of task-relevant concepts can be identified before model development. In this study, these concepts were derived from the MedWeb task documentation and include type of evidence, timing of occurrence, recovery status, and uncertainty of expression (Table 1). The objective is to guide the fine-tuning process such that symptom classification is learned as a function of these concepts.

To implement CALF, the training data is first annotated with respect to the identified concepts by prompting an LLM. In this case, the attributes are treated as categorical variables. Next, the annotated dataset is augmented with counterfactual examples. Each counterfactual is generated by prompting the language model to minimally edit an observed text by modifying the symptom label and one or more associated attributes, while preserving all other content as much as possible. For example, the original text “I have a terrible cough” can be transformed into “I had a terrible fever this morning” by switching the symptom and specifying a recent time of occurrence. During generation, the model is instructed to maximize semantic similarity and restrict changes to specified attributes.

Then, the model is fine-tuned using the counterfactually augmented data. In standard supervised fine-tuning, the training objective is to minimize the multilabel binary cross-entropy loss:


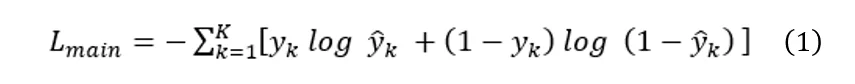


where *y* ∈ {0,1}*^K^* denotes the ground-truth label vector for symptoms, and *ŷ* ∈ [0,1]*^K^* represents the model’s predicted probabilities. However, in the case of CALF, a multitask learning framework is applied. Suppose there are *T* additional attributes. The multitask training objective is:


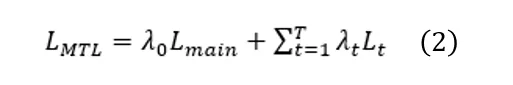


where *λ_0_* and *λ_t_* are task-specific weights, *L_main_* is the loss of the main task in equation 1, and *L_t_* denotes the cross-entropy loss for auxiliary attribute *z_t_*.

Symptom detection is treated as the primary task, while attribute-based classification tasks serve as auxiliary objectives. Joint optimization of these objectives encourages the model to learn representations aligned with surveillance-relevant concepts, thereby improving robustness and attribute sensitivity. The tuning parameters were set to (*λ_0_*=1 and *λ_t_*_=_0.05).

The auxiliary-task weight *λ_t_* was selected using a held-out validation set. Specifically, 20% of the original training data were reserved for validation, while the remaining 80% were used for model training. We evaluated *λ_t_*=∈{0.05, 0.10, 0.15, 0.20} under the corresponding data-augmentation strategy and selected the value that achieved the highest macro *F*_1_ (m-*F*_1_)-score on the validation set. Based on this procedure, the value (*λ_t_*_=_0.05) was selected. The final model was then fine-tuned using the complete training set and the selected weight. The same tuning procedure was applied to all baseline methods that involve a multitask learning objective.

#### Manual Validation of LLM-Generated Annotation

Manual validation of the LLM’s ability to annotate attributes was conducted to ensure the plausibility of the method. The LLM that was tested is GPT-4.1. To assess the quality of generated attribute labels, the author manually audited 150 training instances. The researcher agreed with all GPT-generated labels in 115 of 150 cases (76.7%). At the attribute level, agreement was observed in 141 of 150 cases (94%) for type of evidence, 147 of 150 cases (98%) for recovery status, 143 of 150 cases (95.3%) for timing of occurrence, and 131 of 150 cases (87.3%) for uncertainty of expression. Uncertainty was the most interpretation-sensitive attribute, as speculative, doubtful, conditional, or denial-related language could sometimes be inferred from context despite ambiguous surface wording. Overall, the audit indicated that GPT-4.1 produced sufficiently reliable auxiliary labels for use in the multitask learning framework.

### Baseline Methods

#### Standard Supervised Fine-Tuning (Baseline)

This serves as an experimental control. No data-level or model-level interventions are applied. The pretrained model is fine-tuned on the original MedWeb training dataset. The training objective is to minimize equation 1.

#### Data-Level Interventions

Data-level interventions aim to improve model robustness through training data augmentation. The augmented dataset was then used to fine-tune the pretrained model using the same objective function defined in equation 1. Since data augmentation is ideally a simple and cost-effective alternative to collecting additional annotated data [54], prompt-based approaches were adopted as baseline strategies. The evaluated data-level methods include:

- Backtranslation: Data are augmented by translation-based paraphrasing via English-Japanese-English translation [55,56]. The final step also instructs the LLM to paraphrase the final output.
- Few-shot prompt-based generative data augmentation (Few-Shot Gen): An LLM is prompted to generate synthetic data based on randomly sampled, labeled training examples [57,58].
- Multiattribute generative data augmentation (MGenDA): In addition to few-shot examples, explicit attribute-level constraints are included in the prompt when generating synthetic data. This is motivated by previous work on controllability of data generation [59].
- Multiattribute counterfactual data augmentation (MCouDA): This is the data augmentation step in CALF. Synthetic data are generated by editing existing examples. The symptom label and relevant attributes are changed without generating entirely new texts. A comparison of MGenDA and MCouDA is illustrated in Figure 2.

**Figure 2:**
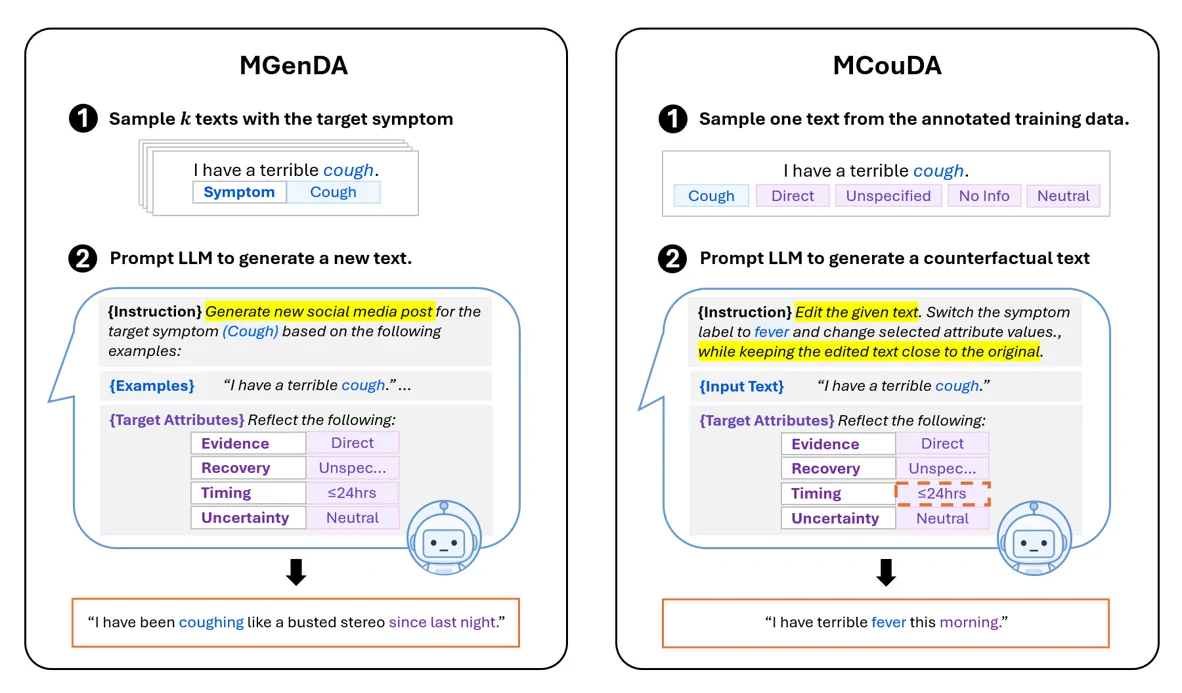
Comparison of multiattribute generative data augmentation (MGenDA) and multiattribute counterfactual data augmentation (MCouDA). Both methods use a large language model (LLM) to generate synthetic training examples while controlling for surveillance-relevant attributes. MGenDA first randomly selects a target symptom and a set of attribute values. The LLM then generates a new post satisfying these conditions, using sampled posts as guidance. MCouDA instead generates a counterfactual text by minimally editing an existing training post so that the symptom label and selected attributes change while the broader context is preserved. The generated examples are then added to the training data. The prompt components shown in the figure are simplified illustrations of the generation process and do not reproduce the full prompts verbatim.

#### Model-Level Intervention: Multitask Learning

Fine-tuning via a multitask learning framework (MTL-only) is evaluated as a model-level–only intervention. In this setting, GPT-4.1 is first used to annotate the training data with surveillance-relevant attributes (Table 1). The main task is symptom detection, while auxiliary tasks correspond to attribute-based classification. The training objective is to minimize equation 2. Similarly, the hyperparameters were set to (*λ_0_*=1, *λ_t_*_=_0.05) as it yielded better results. In this case, no synthetic data were added to the training data.

#### Alternative Hybrid Methods

To further contextualize the design choices of CALF, we evaluated alternative data augmentation and model-level interventions in different ways. First, we considered a hybrid approach in which multiattribute generative data augmentation replaces the CDA, while retaining the multitask learning objective. Second, we explored a hybrid approach based on adapter fusion (AF) [60], where task-specific adapters are trained separately for symptom detection and attribute prediction, and then fusion layers are trained for the final classifier. Unlike typical joint optimization, this approach treats each task independently and combines them post hoc, providing a contrasting hybrid strategy. For the adapter fusion case, both generative data augmentation and CDA were tested. In summary, the alternative hybrid methods are as follows:

- MGenDA+MTL: multiattribute generative data augmentation with multitask learning training objective.
- MCouDA+AF: multiattribute CDA with adapter fusion.
- MGenDA+AF: multiattribute generative data augmentation with adapter fusion.

#### Prompt-Based Methods

In addition to fine-tuned encoder-based models, we evaluated 2 prompt-based baselines in which an LLM was directly instructed to identify symptoms in social media text. Specifically, we evaluated prompting GPT-4.1 (Prompt GPT) and prompting Qwen2.5-7B-Instruct (Prompt Qwen). The prompt was adapted from previous work that also evaluated symptom detection using the MedWeb dataset [12].

### Evaluation Metrics

Model performance was evaluated along two complementary dimensions: (1) classification accuracy on the standard MedWeb test set, (2) robustness to attribute-specific perturbations using the MedWeb checklist dataset, and (3) potential generalization to other data using the supplementary test set based on SPEED++ and MedNLP-SC. Together, these evaluations help verify the research hypotheses.

#### Classification Accuracy

Classification performance on the MedWeb test set was measured using standard multilabel metrics. Each symptom *k* ∈ {1, … , 8} was treated as a binary classification task. Let *TP_k_*, *FP_k_*, and *FN_k_* denote the number of true positives, false positives, and false negatives for symptom *k*, respectively, based on predictions *ŷ_i,k_*≥0.5. Precision and recall are defined as:


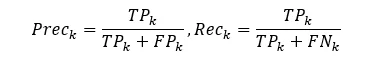


and the per-symptom *F*_1_-score is:


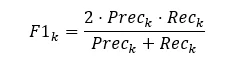


Performance across the 8 symptoms is summarized using the macro-average:


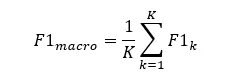


which assigns equal weight to each symptom and prevents frequent symptoms from dominating the evaluation.

#### Robustness to Attribute-Specific Perturbations

Robustness is defined as a model’s ability to respond appropriately to controlled, attribute-specific changes in the input text. The MedWeb checklist dataset enables this evaluation by providing sets of minimally contrasting counterfactual texts that differ only with respect to a single attribute.

For each contrasting pair, a prediction is counted as correct only if the model predicts the correct labels for both texts in the pair. Because each pair is constructed so that the manipulated attribute includes a label change (eg, no evidence vs direct evidence, past case vs present case, and in denial vs speculative), this criterion explicitly evaluates whether the model is sensitive to the edited attribute. We refer to this metric as flip accuracy (FA). Formally, to evaluate sensitivity to attribute j for symptom k, the FA is computed as:


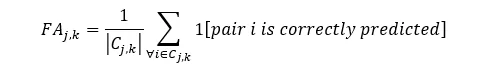


where *C_j,k_* denotes the set of contrasting pairs for attribute *j* and symptom *k*, and 1[.] is an indicator function equal to 1 when both labels in the pair are correctly predicted.

Overall robustness is summarized by macro-averaging FA across attributes, symptoms, or both, reflecting the expectation that a robust model should behave consistently across different symptom categories and attribute-specific perturbations.

#### Statistical Analysis of Evaluation Metrics

We used nonparametric bootstrap-based inference to estimate uncertainty in the evaluation metrics and test whether each method outperformed the standard fine-tuning baseline [61,62]. Resampling within strata preserved the distribution of categorical case characteristics, reduced the risk of omitting underrepresented categories, and maintained common symptom co-occurrence patterns. The same resampled cases were used across methods to enable paired comparisons. We generated (B=1000) bootstrap replicates for each analysis. Percentile-based 95% CIs were also estimated for the evaluation scores and their differences relative to the baseline.

For each method, the observed score difference relative to the baseline is defined as Δ*S*=*S*_Method_–*S*_Baseline_. We conducted a 1-sided bootstrap-based test, comparing *H*_0_: E(Δ*S*)≤0 vs *H*_1_: E(Δ*S*)>0. Let Δ*S_b_* denote the score difference in the *b^th^* bootstrap resample. Then, the null-adjusted bootstrap differences are calculated as:

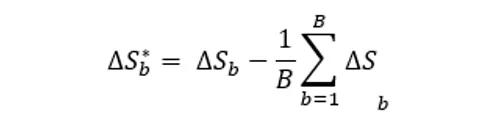



The bootstrap-based *P* value is calculated as:


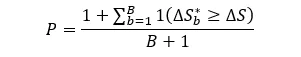


A *P* value lower than .05 was considered sufficient evidence to conclude that the method outperformed the baseline with respect to the said metric and test set.

### Ethical Considerations

This study did not require human or animal participants to undergo any physical or mental interventions. The research did not involve processing personally identifiable information. Only publicly available or deidentified data were used. Accordingly, institutional review board approval was not required under the Ethical Guidelines for Medical and Health Research Involving Human Subjects established by the Japanese government. The MedWeb and MedNLP-SC datasets are distributed under the Creative Commons Attribution 4.0 International License, while the SPEED++ dataset was obtained from its original authors and used exclusively for research purposes. LLMs were accessed through official APIs and used in accordance with their terms of service. Since LLM-based pipelines may involve transmitting text to an external service, future implementations should minimize the transmission of personally identifiable or sensitive information and comply with applicable privacy requirements and institutional policies.

## Results

### Comparison of Evaluation Metrics Across Different Test Sets

Performance score is measured using m-*F*_1_ for the MedWeb test set, overall FA for the MedWeb checklist dataset, and *F*_1_-score for flu-or-fever detection (f-*F*_1_) for the supplementary test set. The relative difference to the baseline score (Δ*S*) is also reported. *P* values (*P*) were estimated using 1-sided paired-bootstrap tests against the standard fine-tuning baseline, with the null hypothesis that the method does not outperform the baseline. A *P* value of .05 or lower indicates a statistically significant improvement over the baseline. Dashes indicate the reference baseline.

Table 2 summarizes the performance of the encoder-based and prompt-based methods across the 3 evaluation datasets. The reported encoder-based results use RoBERTa-large as the pretrained encoder and GPT-4.1 for synthetic data generation or LLM-based annotation, where applicable.

On the MedWeb test set, only Few-Shot Gen (m-*F*_1_=0.886, *P*=.05) achieved significantly higher m-*F*_1_-scores than the standard fine-tuning baseline (m-*F*_1_=0.871). Most encoder-based methods have comparable m-*F*_1_-scores to the baseline. The prompt-based methods achieved lower m-*F*_1_-scores than the baseline. Prompt GPT-4.1 obtained an m-*F*_1_ of 0.776 (95% CI 0.755-0.796), while Prompt Qwen obtained an m-*F*_1_ of 0.755 (95% CI 0.733-0.776).

On the MedWeb checklist dataset, all evaluated methods achieved significantly higher overall FA than the baseline (FA=0.342). Among the evaluated approaches, Prompt GPT-4.1 achieved the highest FA (FA=0.920, *P*=.001), followed by CALF (FA=0.830, *P*=.001) and McouDA-only (FA=0.829, *P*=.001).

On the supplementary test set, MGenDA+MTL (f-*F*_1_=.855, *P*=.03), CALF (f-*F*_1_=0.856, *P*=.007), Prompt GPT-4.1 (f-*F*_1_=0.910, *P*=.001), and Prompt Qwen (f-*F*_1_=0.853, *P*=.05) achieved statistically higher f-*F*_1_-scores than the baseline (f-*F*_1_=0.797). The remaining methods were not significantly different from the baseline.

Among the approaches, MGenDA+MTL and CALF were the only methods that significantly outperformed the baseline across all 3 evaluation datasets. In an exploratory rank-correlation analysis, performance on the MedWeb checklist dataset was strongly associated with performance on the supplementary test set (Spearman ρ=0.811). By contrast, MedWeb test-set performance showed weak negative associations with both checklist performance (ρ=–0.266) and supplementary test performance (ρ=–0.042).

**Table 2:** Performance of encoder-based and prompt-based methods across test datasets.

Method	MedWeb test				MedWeb checklist				Supplementary test		
	m-*F*_1_	Δ*S*	*P* value	FA		Δ*S*	*P* value	f-*F*_1_		Δ*S*	*P* value
Baseline	0.871	—^a^	—	0.342		—	—	0.797		—	—
Backtranslation	0.881	0.009	.13	0.363		0.021	.003	0.796		–0.001	.52
MTL-only^b^	0.876	0.004	.22	0.383		0.041	.001	0.802		0.005	.39
Few-Shot Gen^c^	0.886	0.014	.05^d^	0.565		0.224	.001	0.811		0.015	.29
MGenDA^e^-only	0.883	0.012	.09	0.776		0.434	.001	0.834		0.037	.08
MCouDA^f^-only	0.865	–0.007	.78	0.829		0.487	.001	0.806		0.009	.36
MGenDA+AF^g^	0.827	–0.044	≥.99	0.751		0.410	.001	0.831		0.034	.12
MCouDA+AF^h^	0.827	–0.044	≥.99	0.724		0.383	.001	0.792		–0.005	.59
MGenDA+MTL^i^	0.882	0.010	.16	0.821		0.479	.001	0.855		0.059	.03
CALF^j^	0.880	0.009	.18	0.830		0.488	.001	0.856		0.059	.007
Prompt GPT^k^	0.776^l^	–0.096	≥.99	0.920		0.578	.001	0.910		0.113	.001
Prompt Qwen^m^	0.755^l^	–0.116	≥.99	0.780		0.439	.001	0.853		0.056	.05

^a^Not applicable.

^b^MTL: fine-tuning via multitask learning framework.

^c^Few-Shot Gen: few-shot prompt-based generative data augmentation.

^d^The exact *P* value is .04995.

^e^MgenDA: multiattribute generative data augmentation.

^f^MCouDA: multiattribute counterfactual data augmentation.

^g^MGenDA+AF: multiattribute generative data augmentation with adapter fusion.

^h^MCouDA+AF: multiattribute counterfactual data augmentation with adapter fusion.

^i^MGenDA+MTL: multiattribute generative data augmentation with multitask learning training objective.

^j^CALF: concept-aware learning framework.

^k^Prompt GPT: prompting GPT-4.1 for symptom classification task.

^l^The 95% CI estimate for the relative difference to the baseline also indicates that the method performs statistically weaker than the baseline.

^m^Prompt Qwen: prompting Qwen2.5-7B-Instruct for symptom classification task.

### Attribute Level Analysis

Table 3 presents FA by surveillance-relevant attribute. The baseline achieved high scores for direct evidence (FA=0.838) and indirect evidence (FA=0.607), but substantially lower scores for recovery status (FA=0.077), timing of occurrence (FA=0.059), and uncertainty of expression (FA=0.127). Relative to the baseline, prompt-based methods achieved substantially better FA for each attribute. Prompt GPT achieved scores that are consistently within the top 3 per attribute (FA range=0.867-0.961). Prompt Qwen also substantially improved the FA per attribute relative to the baseline. However, compared with Prompt GPT, the FA of Prompt Qwen is much lower for timing (FA=0.777 vs 0.951) and uncertainty (FA=0.434 vs 0.867).

In terms of encoder-based models, Backtranslation only improved sensitivity of direct evidence (FA=0.909) and indirect evidence (FA=0.728), while the remaining attributes have an FA below 0.10. Similarly, MTL-only achieved substantially better scores in 2 attributes, recovery (FA=0.114) and uncertainty (FA=0.277), but the improvement is minimal. Few-Shot Gen achieved statistically better scores than the baseline in 4 attributes, but sensitivity to uncertainty (FA=0.192) remains low. In contrast, methods with multiattribute control (MGenDA or MCouDA) consistently and substantially improved the baseline score for recovery (FA=0.702 or higher), timing (FA=0.464 or higher), and uncertainty (FA=0.678 or higher). Moreover, MCouDA only, and CALF achieved a statistically higher FA than the baseline in all attributes.

**Table 3:** Per attribute, flip accuracy by training method. The table reports flip accuracy for each surveillance-related attribute. For encoder-based models, RoBERT^a^-Large is the base model, and GPT-4.1 is used for data augmentation.

Method	Overall flip accuracy				
	Direct	Indirect	Recovery	Timing	Uncertainty
Baseline	0.838	0.607	0.077	0.059	0.127
Backtranslation	0.909^a^	0.727^a^	0.056	0.046	0.078
MTL^b^-only	0.853	0.604	0.114^a^	0.065	0.277^a^
Few-Shot Gen^c^	0.746	0.697^a^	0.450^a^	0.740^a^	0.192^a^
MGenDA^d^-only	0.833	0.731^a^	0.779^a^	0.852^a^	0.684^a^
MCouDA^e^-only	0.871^a^	0.708^a^	0.786^a^	0.918^a^	0.864^a^
MGenDA+AF^f^	0.843	0.627	0.783^a^	0.826^a^	0.678^a^
MCouDA+AF^g^	0.913^a^	0.594	0.856^a^	0.464^a^	0.795^a^
MGenDA+MTL^h^	0.856	0.731^a^	0.855^a^	0.870^a^	0.791^a^
CALF^i^	0.877^a^	0.670^a^	0.824^a^	0.914^a^	0.862^a^
Prompt GPT^j^	0.912^a^	0.909^a^	0.961^a^	0.951^a^	0.867^a^
Prompt Qwen^k^	0.926^a^	0.860^a^	0.915^a^	0.777^a^	0.434^a^

^a^Assuming a 5% level of significance, there is sufficient evidence to conclude that the result is statistically better than the baseline.

^b^MTL: fine-tuning via multitask learning framework.

^c^Few-Shot Gen: few-shot prompt-based generative data augmentation.

^d^MgenDA: multiattribute generative data augmentation.

^e^MCouDA: multiattribute counterfactual data augmentation.

^f^MGenDA+AF: multiattribute generative data augmentation with adapter fusion.

^g^MCouDA+AF: multiattribute counterfactual data augmentation with adapter fusion.

^h^MGenDA+MTL: multiattribute generative data augmentation with multitask learning training objective.

^i^CALF: concept-aware learning framework.

^j^Prompt GPT: prompting GPT-4.1 for symptom classification task.

^k^Prompt Qwen: prompting Qwen2.5-7B-Instruct for symptom classification task.

### Symptom Level Analysis

Figure 3 presents per-symptom *F*_1_-scores on the MedWeb test set. Performance varied across symptom categories and methods. The encoder-based approaches generally produced more consistent per-symptom *F*_1_-scores than the prompt-based methods. For encoder-based methods, the per-symptom *F*_1_-scores are at least 0.75 across all symptoms except for influenza, while both prompt-based methods included have 2 to 4 symptoms with *F*_1_-scores below 0.75. Also, in the case of Prompt GPT, the *F*_1_-score for influenza is below 0.50.

Table 4 presents the FA separately for each symptom. The baseline achieved low FA scores across all symptoms, ranging from FA=0.26 for influenza to FA=0.41 for cold. Compared with the baseline, Prompt GPT achieved high FA scores in all symptoms, ranging from FA=0.83 for cold to FA=0.96 for headache. Prompt Qwen also significantly outperformed the baseline across all symptoms, although its scores were consistently lower than those of Prompt GPT.

Similar to the per-attribute results, the improvements associated with Backtranslation, MTL-only, and Few-Shot Gen were limited or uneven across symptom categories. In contrast, MGenDA only, MCouDA only, MGenDA+AF, MCouDA+AF, MGenDA+MTL, and CALF significantly outperformed the baseline for all 8 symptoms, although the magnitude of improvement varied by method and symptom.

Among the encoder-based approaches, MCouDA only and CALF showed the strongest worst-case performance; both achieved a minimum per-symptom FA of 0.75 for cold. This minimum exceeded the lowest score observed for Prompt Qwen (FA=0.66 for cold), but remained below the lowest score achieved by Prompt GPT-4.1 (FA=0.83 for cold). McouDA-only and CALF also ranked among the strongest encoder-based methods across most symptom categories.

**Figure 3:**
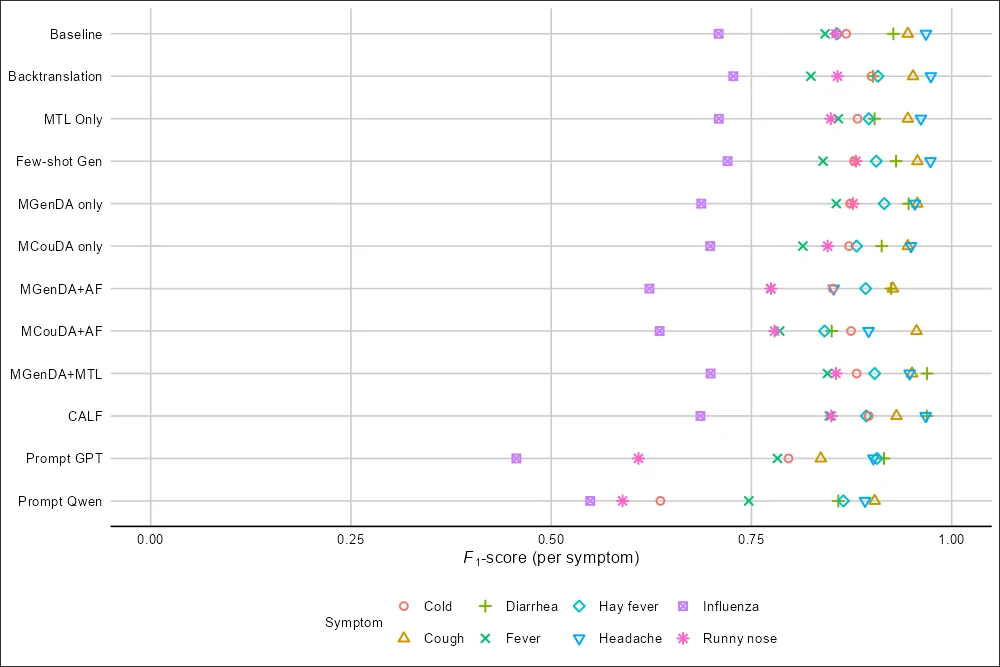
Scatterplot of per-symptom F1-scores by method. Each point represents the F1-score of the method for certain symptom or disease. CALF: concept-aware learning framework; Few-Shot Gen: few-shot prompt-based generative data augmentation; MCouDA: multiattribute counterfactual data augmentation; MCouDA+AF: multiattribute counterfactual data augmentation with adapter fusion; MGenDA: multiattribute generative data augmentation; MGenDA+AF: multiattribute generative data augmentation with adapter fusion; MGenDA+MTL: multiattribute generative data augmentation with multitask learning training objective; MTL: fine-tuning via multitask learning framework; Prompt GPT: prompting GPT-4.1 for symptom classification task; Prompt Qwen: prompting Qwen2.5-7B-Instruct for symptom classification task.

**Table 4:** Per symptom, flip accuracy by method. The table reports flip accuracy for each symptom category. For encoder-based methods, RoBERT^a^-large was used as the pretrained encoder and GPT-4.1 was used for synthetic-data generation, where applicable.

Method	Per symptom, flip accuracy							
	Cold	Cough	Diarrhea	Fever	Hay fever	Headache	Influenza	Runny nose
Baseline	0.41	0.39	0.27	0.38	0.33	0.31	0.26	0.39
Backtranslation	0.42	0.39	0.28	0.36	0.32	0.32	0.43^a^	0.40
MTL^b^-only	0.43	0.40	0.33^a^	0.42^a^	0.35	0.36^a^	0.34^a^	0.43^a^
Few-Shot Gen^c^	0.58^a^	0.70^a^	0.61^a^	0.64^a^	0.21	0.54^a^	0.62^a^	0.63^a^
MGenDA^d^-only	0.69^a^	0.86^a^	0.75^a^	0.82^a^	0.82^a^	0.67^a^	0.78^a^	0.81^a^
MCouDA^e^-only	0.75^a^	0.90^a^	0.79^a^	0.88^a^	0.84^a^	0.80^a^	0.82^a^	0.85^a^
MGenDA+AF^f^	0.71^a^	0.84^a^	0.71^a^	0.82^a^	0.79^a^	0.70^a^	0.63^a^	0.81^a^
MCouDA+AF^g^	0.70^a^	0.83^a^	0.64^a^	0.71^a^	0.81^a^	0.74^a^	0.57^a^	0.80^a^
MGenDA+MTL^h^	0.74^a^	0.93^a^	0.77^a^	0.90^a^	0.82^a^	0.76^a^	0.81^a^	0.84^a^
CALF^i^	0.75^a^	0.93^a^	0.76^a^	0.88^a^	0.80^a^	0.83^a^	0.77^a^	0.91^a^
Prompt GPT^j^	0.83^a^	0.95^a^	0.90^a^	0.95^a^	0.92^a^	0.96^a^	0.94^a^	0.91^a^
Prompt Qwen^k^	0.66^a^	0.85^a^	0.81^a^	0.84^a^	0.75^a^	0.81^a^	0.77^a^	0.75^a^

^a^Assuming a 5% level of significance, there is sufficient evidence to conclude that the result is statistically better than the baseline.

^b^MTL: fine-tuning via multitask learning framework.

^c^Few-Shot Gen: few-shot prompt-based generative data augmentation.

^d^MgenDA: multiattribute generative data augmentation.

^e^MCouDA: multiattribute counterfactual data augmentation.

^f^MGenDA+AF: multiattribute generative data augmentation with adapter fusion.

^g^MCouDA+AF: multiattribute counterfactual data augmentation with adapter fusion.

^h^MGenDA+MTL: multiattribute generative data augmentation with multitask learning training objective.

^i^CALF: concept-aware learning framework.

^j^Prompt GPT: prompting GPT-4.1 for symptom classification task.

^k^Prompt Qwen: prompting Qwen2.5-7B-Instruct for symptom classification task.

### Sensitivity to the Choice of Language Models

Figure 4 presents overall checklist FA across pretrained encoders and LLMs used for synthetic-data generation. Baseline performance varied substantially across encoders. FA was highest for BERTweet-large (FA=0.399, 95% CI 0.379-0.419), followed by RoBERTa-large (FA=0.342, 95% CI 0.325-0.360), BioMed-RoBERTa (FA=0.222, 95% CI 0.208-0.235), and ClinicalBERT (FA=0.151, 95% CI 0.137-0.164). Across the evaluated methods, BERTweet-large and RoBERTa-large generally achieved higher scores than BioMed-RoBERTa and ClinicalBERT.

All evaluated data-augmentation and hybrid methods improved FA relative to their corresponding encoder-specific baselines. When GPT-4.1 was used for synthetic-data generation, CALF achieved the highest FA for each pretrained encoder: BERTweet-large (FA=0.842, 95% CI 0.822-0.861), RoBERTa-large (FA=0.830, 95% CI 0.808-0.851), BioMed-RoBERTa (FA=0.732, 95% CI 0.710-0.755), and ClinicalBERT (FA=0.587, 95% CI 0.565-0.609).

When Qwen2.5-7B-Instruct was used for synthetic-data generation, the highest-performing method varied across encoders. CALF achieved the highest Qwen-based score for BERTweet-large (FA=0.738). For RoBERTa-large, MGenDA+MTL achieved the highest score (FA=0.764), followed by CALF (FA=0.737). For ClinicalBERT, MGenDA+MTL also achieved the highest score (FA=0.436), compared with FA=0.386 for CALF. For BioMed-RoBERTa, MgenDA-only achieved the highest score (FA=0.564), while CALF achieved a similar score (FA=0.555).

For BERTweet-large and RoBERTa-large, the hybrid methods generally achieved higher scores than their corresponding augmentation-only counterparts. With GPT-4.1, MGenDA+MTL improved upon MGenDA-only for BERTweet-large (0.811 vs 0.778) and RoBERTa-large (0.821 vs 0.776). CALF achieved numerically similar scores to MCouDA-only for BERTweet-large (0.842 vs 0.841) and RoBERTa-large (0.830 vs 0.829). With Qwen2.5-7B-Instruct, MGenDA+MTL also outperformed MGenDA-only for BERTweet-large (0.731 vs 0.725) and RoBERTa-large (0.764 vs 0.728), while CALF outperformed MCouDA-only for BERTweet-large (0.738 vs 0.708) and RoBERTa-large (0.737 vs 0.653).

**Figure 4:**
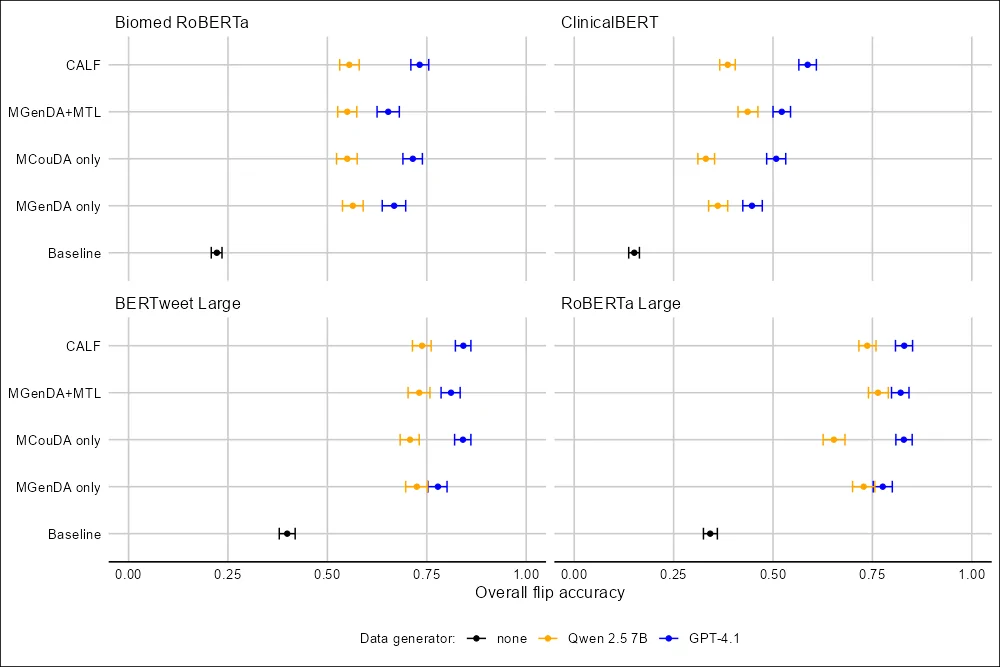
Overall flip accuracy by training method and pretrained model. The figure shows the overall flip accuracy estimates, and the error bands are based on the corresponding 95% CI. The plot shows the result for using GPT-4.1 and Qwen2.5-7B-instruct for label annotation and synthetic data generation. CALF: concept-aware learning framework; MCouDA: multiattribute counterfactual data augmentation; MGenDA: multiattribute generative data augmentation; MGenDA+MTL: multiattribute generative data augmentation with multitask learning training objective.

Figure 5 presents f-*F*_1_-scores on the supplementary test set across pretrained encoders and LLMs used for synthetic-data generation. Similar to checklist performance, supplementary test scores were generally higher for BERTweet-large and RoBERTa-large than for BioMed-RoBERTa and ClinicalBERT. However, the differences between GPT-4.1–based and Qwen-based generation were smaller for MGenDA only and MGenDA+MTL than for McouDA-only and CALF. For McouDA-only and CALF, GPT-4.1–based generation generally produced higher supplementary test scores than Qwen-based generation.

Figure 6 presents the relationship between overall checklist FA and supplementary-test *F*_1_-score. Based on the plot, methods with higher checklist FA tend to achieve higher supplementary test scores. The 2 metrics showed a strong positive Spearman rank correlation (ρ=0.849).

Table 5 presents per-attribute FA for BERTweet-large and RoBERTa-large under different synthetic data generation settings. For both encoders, the baseline achieved relatively high scores for direct and indirect evidence but substantially lower scores for recovery status, timing of occurrence, and uncertainty of expression. In addition, the training strategies included consistently produced substantial improvements in these 3 attributes under both GPT-4.1–based and Qwen-based generation.

For RoBERTa-large, CALF significantly outperformed the corresponding baseline across all attributes under both GPT-4.1–based and Qwen-based generation. For BERTweet-large, CALF also achieved high scores across attributes, although the improvement for indirect evidence was less consistent. The largest GPT-4.1 versus Qwen differences were observed for recovery status and uncertainty of expression. For example, with BERTweet-large, CALF achieved FA=0.844 for recovery and FA=0.888 for uncertainty under GPT-4.1–based generation, compared with FA=0.583 and FA=0.693, respectively, under Qwen-based generation.

**Figure 5:**
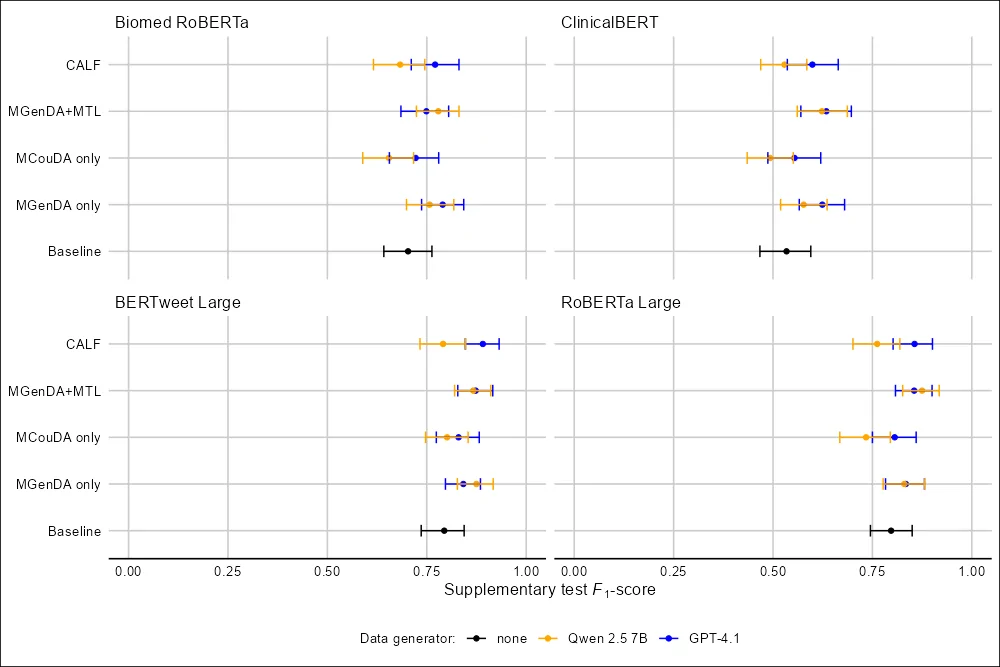
Supplementary test F1-score by training method and pretrained model. The figure shows the flu-or-fever F1-score estimates, and the error bands are based on the corresponding 95% CI. The plot shows the result for using GPT-4.1 and Qwen2.5-7B-instruct for label annotation and synthetic data generation. CALF: concept-aware learning framework; MCouDA: multiattribute counterfactual data augmentation; MGenDA: multiattribute generative data augmentation; MGenDA+MTL: multiattribute generative data augmentation with multitask learning training objective.

**Figure 6:**
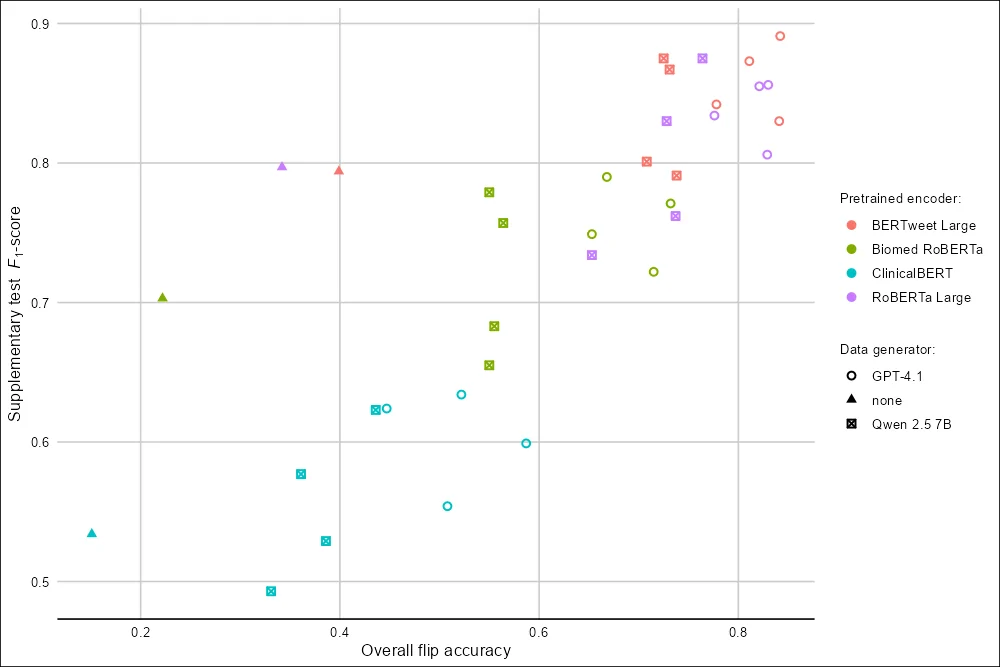
Scatterplot of supplementary test F1-score and overall flip accuracy. Each point represents a score for a method. For baseline models, the data generator is “none”.

**Table 5:** Per attribute, flip accuracy by training method, data generator, and pretrained encoder. The table reports flip accuracy for each surveillance-related attribute.

Encoder, generator, and method			Per attribute, flip accuracy				
			Direct evidence	Indirect evidence	Recovery	Timing	Uncertainty
**BERTweet**							
	**GPT-4.1**						
		MGenDA^a^-only	0.843	0.724^b^	0.734^b^	0.853^b^	0.737^b^
		MCouDA^c^-only	0.885^b^	0.716^b^	0.811^b^	0.912^b^	0.880^b^
		MGenDA+MTL^d^	0.856	0.739^b^	0.796^b^	0.878^b^	0.785^b^
		CALF^e^	0.893^b^	0.665	0.844^b^	0.919^b^	0.888^b^
	**Qwen 2.5 7B**						
		MGenDA-only	0.867	0.665	0.502^b^	0.832^b^	0.675^b^
		MCouDA-only	0.774	0.671	0.635^b^	0.797^b^	0.747^b^
		MGenDA+MTL	0.802	0.663	0.696^b^	0.782^b^	0.711^b^
		CALF	0.896^b^	0.671	0.583^b^	0.848^b^	0.693^b^
	**None**						
		Baseline	0.853	0.641	0.137	0.170	0.193
**RoBERTa**							
	**GPT-4.1**						
		MGenDA-only	0.833	0.731^b^	0.779^b^	0.852^b^	0.684^b^
		MCouDA-only	0.871^b^	0.708^b^	0.786^b^	0.918^b^	0.864^b^
		MGenDA+MTL	0.856	0.731^b^	0.855^b^	0.870^b^	0.791^b^
		CALF	0.877^b^	0.670^b^	0.824^b^	0.914^b^	0.862^b^
	**Qwen 2.5 7B**						
		MGenDA-only	0.804	0.669^b^	0.740^b^	0.766^b^	0.661^b^
		MCouDA-only	0.848	0.607	0.535^b^	0.666^b^	0.608^b^
		MGenDA+MTL	0.835	0.706^b^	0.746^b^	0.796^b^	0.736^b^
		CALF	0.903^b^	0.656^b^	0.646^b^	0.771^b^	0.709^b^
	**None**						
		Baseline	0.838	0.607	0.077	0.059	0.127

^a^MGenDA: multiattribute generative data augmentation.

^b^Assuming a 5% level of significance, there is sufficient evidence to conclude that the result is statistically better than the corresponding baseline.

^c^MCouDA: multiattribute counterfactual data augmentation.

^d^MGenDA+MTL: multiattribute generative data augmentation with multitask learning training objective.

^e^CALF: concept-aware learning framework.

## Discussion

### Principal Findings

Results show that models may perform well on held-out data while remaining insufficiently sensitive to attributes that should influence symptom detection. The standard fine-tuning baseline performed well on the MedWeb test set, but the MedWeb checklist revealed limited sensitivity to timing of occurrence, recovery status, and uncertainty of expression. By contrast, the baseline responded more reliably to direct and indirect symptom evidence. This pattern appeared for both BERTweet and RoBERTa. These findings suggest that held-out performance may be driven largely by the ability to recognize symptom mentions rather than interpret the context in which they appear. Stronger MedWeb test performance also did not consistently translate into better supplementary test performance. Backtranslation has a slightly higher m-*F*_1_-score on the held-out test set but performed slightly worse than the baseline on the supplementary test. Similarly, Few-Shot Gen achieved the highest MedWeb test score but did not rank among the strongest methods on the supplementary test. Thus, conventional held-out metrics alone provide limited evidence of out-of-distribution generalizability. In some cases, improvements may reflect stronger fitting to patterns already represented in the training data. The Backtranslation results support this interpretation. Its improvement in FA were concentrated in direct and indirect evidence, which were already the strongest attributes for the baseline.

Checklist performance may provide a more informative signal of potential generalizability. Methods that performed well on the MedWeb checklist also tended to achieve stronger supplementary test results, whereas MedWeb test performance showed weak negative association with either checklist or supplementary test performance. This pattern remained visible across the different encoders and data generators. Sensitivity to surveillance-relevant attributes may therefore offer useful information beyond conventional held-out metrics. However, this interpretation remains preliminary because the supplementary set contains only 200 curated examples and focuses on influenza-or-fever detection. Larger external evaluations covering more symptoms are needed to determine whether checklist performance consistently predicts out-of-distribution performance.

Incorporating surveillance-relevant concepts into training addressed several weaknesses of standard fine-tuning. CALF improved sensitivity to recovery status, timing of occurrence, and uncertainty of expression, which were the weakest attributes for the baseline. These improvements appeared across different encoders and under both GPT- and Qwen-based augmentation. CALF also provided one of the strongest and most balanced encoder-based results across the MedWeb test set, checklist dataset, and supplementary test. Under the primary setting using RoBERTa and GPT, CALF substantially improved performance on the MedWeb checklist and supplementary test sets while achieving a slightly higher score than standard fine-tuning on the MedWeb test set. CALF also frequently achieved the highest score among encoder-based methods when GPT was used for data augmentation. However, it was not uniformly superior across all settings. When Qwen was used, MGenDA+MTL sometimes achieved comparable or slightly stronger results. These findings support CALF as a robust encoder-based strategy while indicating that its relative advantage depends partly on the LLM used for data augmentation.

The strongest encoder-based results generally came from hybrid methods. Augmentation methods that explicitly controlled multiple attributes produced broader improvements in recovery, timing, and uncertainty than Backtranslation or Few-Shot Gen. Moreover, MGenDA+MTL and CALF generally outperformed their augmentation-only counterparts, MGenDA-only and MCouDA-only, when paired with stronger encoders. This pattern suggests that data-level and model-level interventions play complementary roles. Controlled augmentation exposes the model to relevant attribute combinations, while multitask learning encourages the encoder to represent those attributes during classification.

Generator quality may be particularly important for counterfactual augmentation. MCouDA and CALF require the LLM to preserve the original context while modifying selected symptoms and attributes. This constrained editing task may be more difficult than the open-ended generation used in MGenDA because several conditions must be satisfied at once. In a preliminary qualitative audit of 30 counterfactuals per generator, GPT produced more clearly acceptable outputs than Qwen (22 of 30 vs 18 of 30). Some Qwen examples introduced implausible context changes. For instance, “Those who have to cough should wear a mask, it’s good manners” became “Those who have to experience diarrhea should wear a mask, it’s good manners.” Since the advantage of GPT was also observed on the supplementary test set, the stronger performance cannot be attributed solely to LLM-specific artifacts. Although the audit was limited in size, the findings suggest that counterfactual quality may partly explain why CALF showed a clearer advantage when GPT was used.

Finally, the prompt-based methods showed a distinct performance profile. Direct prompting of GPT and Qwen produced strong checklist and supplementary-test performance without task-specific fine-tuning. Because these models were not trained on the MedWeb training set, they may be less influenced by correlations specific to that dataset. Their checklist results also indicate that instruction-following LLMs can respond appropriately to controlled attribute changes. However, both prompt-based methods performed less strongly on the original MedWeb test set than the encoder-based approaches. Preliminary inspection suggests that many disagreements involved posts with multiple symptoms or labels that depend on dataset-specific annotation conventions. For example, in “I got the flu and everyone in my club laughed at me,” GPT correctly identified influenza but did not predict fever, although influenza-positive cases in MedWeb are also labeled positive for fever. In another example, “My nose is runny. I feel out of it and my head’s started to hurt. Probably headache medicine will work better than cold medicine,” GPT identified cold in addition to headache and runny nose, likely because the post explicitly mentions cold medicine. These predictions are plausible from the text but do not fully match the MedWeb labels. This suggests that some apparent errors may reflect annotation conventions or artifacts of dataset construction rather than a failure to interpret symptom-related context. A larger error analysis is needed to confirm this explanation.

Direct prompting offers a simple and robust baseline when access to capable LLMs is available. However, repeated inference with large proprietary models may be costly or unsuitable when text cannot be sent to an external service. An alternative is to use an LLM during model development to generate concept-aware training data, then deploy a smaller encoder locally. With suitable encoder and generator choices, CALF and related hybrid methods can provide strong robustness while retaining the efficiency and accessibility of encoder-based models.

### Error Analysis

This section summarizes the common error patterns observed in the baseline model and illustrates how these errors are reduced when training with CALF. Across both controlled counterfactual evaluations (MedWeb checklist) and real-world COVID-19 data (SPEED++), the baseline model exhibits a consistent reliance on surface-level symptom cues, while the CALF model demonstrates improved sensitivity to sensitivity to surveillance-relevant attributes such as recovery status, timing of occurrence, and uncertainty of expression. A more detailed analysis is included in Multimedia Appendix 3.

In the MedWeb checklist data, 3 recurring error patterns emerge:

- Failure to distinguish recovered vs ongoing symptoms: The baseline model frequently predicts positive labels even when symptoms are explicitly described as fully resolved. For example, in “I went on a trip and brought back a headache as a souvenir, but it’s completely gone now,” the baseline predicts positive for headache, whereas CALF correctly predicts a negative label.
- Misinterpretation of symptom timing: The baseline model does not differentiate symptoms with respect to timing of occurrence. Symptoms that occurred days, weeks, or months ago are still detected as a positive case. In “Last week, during allergy season, I had a persistent cough,” the baseline predicts positive for cough, even though the symptom clearly occurred in the past. CALF instead correctly predicts negative for cough.
- Overprediction under speculation: While expression of in denial is ideally considered as a positive case, hypothetical and speculative expressions should not be predicted as positive. The baseline often misclassifies speculative expressions of a symptom. For example, in “If I go out, I’m worried I might get a fever,” the baseline predicts positive for fever, while CALF correctly predicts a negative label.

Similar patterns are observed in the SPEED++ dataset, which consists of real-world COVID-19 social media posts and serves as an out-of-distribution evaluation.

- Sensitivity to indirect evidence: Although MedWeb contains no COVID-19–related training data, CALF sometimes infers symptoms such as fever from explicit mentions of COVID-19 infection. In “My cousin has tested positive for #(CoronaVirus),” CALF predicts positive for fever while the baseline detects no symptom. This behavior suggests improved contextual reasoning but may also introduce errors when explicit evidence is absent or contradictory.
- Informational or advisory posts misclassified as cases: The baseline model frequently detects symptoms in informational or advisory posts, despite the absence of self-reported illness. CALF is less prone to this error. For example, the baseline detects cough in “GET TEST for COVID-19 Even if you have mild symptoms – scratchy throat, cough, runny nose or slight fever…,” while CALF detects no symptom.
- Questions interpreted as symptom reports: The baseline model often assigns positive labels to questions or rhetorical expressions mentioning symptoms, whereas CALF is less likely to detect these as positive cases. For example, in “Did I hear a cough?,” the baseline predicts positive for cough while CALF detects no symptom.

### Limitations

This study has several limitations. First, the checklist dataset, auxiliary labels, and synthetic training examples were generated using LLM-based procedures. This may introduce model-specific linguistic or stylistic artifacts. To reduce this concern, the checklist construction pipeline was separated from the training augmentation pipeline, and a small supplementary test set from SPEED++ and MedNLP-SC was used to examine whether the findings extend beyond LLM-generated checklist examples. However, this supplementary evaluation was exploratory, limited to 200 curated cases, and focused only on influenza-or-fever detection.

Second, although generated counterfactuals and GPT-generated attribute labels underwent quality checks, the validation was limited in scale. We used LLM-based filtering, manual audits, and qualitative error analysis, but larger expert or crowd-sourced validation studies are needed to better quantify label and generation quality.

Third, the checklist was limited to 5 contrast types based on 4 surveillance-relevant attributes: direct evidence, indirect evidence, timing of occurrence, recovery status, and uncertainty of expression. These attributes were selected because they are relevant to the MedWeb annotation guidelines and can be reasonably inferred and controlled from text alone. This does not imply that they are the only surveillance-relevant attributes. The framework can be extended to additional attributes, provided that they are clearly defined, relevant to the task, and can be reliably generated, annotated, and validated.

Finally, the study focused on the English subset of MedWeb and on symptom classification. Results may differ in multilingual settings, larger datasets, or more complex surveillance tasks such as symptom extraction, temporal normalization, geolocation, or user-level aggregation.

### Conclusions

This study shows that strong held-out test performance alone does not establish robustness to dataset-specific or spuriously correlated features in social media symptom detection. Models may identify symptom mentions accurately while remaining insufficiently sensitive to contextual attributes that influence whether a post is relevant for surveillance. The MedWeb checklist complements standard classification metrics by measuring sensitivity to controlled changes in selected attributes: type of evidence, timing of occurrence, recovery status, and uncertainty of expression. Accordingly, FA should be interpreted as a targeted measure of behavioral robustness with respect to these attributes rather than as a comprehensive measure of robustness.

CALF addresses several weaknesses of standard fine-tuning by combining multiattribute CDA with multitask learning. Across the evaluated settings, CALF improved sensitivity to the selected surveillance-relevant attributes and provided one of the strongest and most balanced results among encoder-based methods across the MedWeb test set, checklist dataset, and exploratory supplementary test. Its gains were observed across pretrained encoders and remained visible with both GPT-4.1 and Qwen2.5-7B-Instruct, although the magnitude of improvement depended partly on the synthetic data generator.

The exploratory supplementary evaluation also suggests that checklist performance may provide useful information about potential out-of-distribution performance. Methods with stronger checklist scores tended to perform better on the curated supplementary set, whereas standard MedWeb test set performance showed little association with supplementary test results. However, this evidence remains preliminary because the supplementary set was small and focused on influenza-or-fever detection.

Overall, the findings support the use of targeted behavioral evaluation and concept-aware training for developing symptom detection models that respond more consistently to surveillance-relevant context. The framework can be extended to additional attributes, symptoms, languages, and health-related tasks, provided that the selected concepts are clearly defined, reliably generated, and independently validated. Future work should evaluate the approach on larger external datasets and examine how synthetic data quality affects the value of counterfactual augmentation.

## Data Availability

The MedWeb and MedNLP-SC datasets are publicly available through the NTCIR website. The SPEED++ dataset may be requested directly from the authors of the original study. The MedWeb Checklist Dataset is available in the GitHub repository [63]. Additional documentation, including the prompt templates used in this study, will be added to the repository.

## Appendix

Multimedia Appendix 1 This presents the tables of descriptive statistics about the training, test, and checklist datasets for the MedWeb task.

Multimedia Appendix 2 This is a detailed discussion of construction and quality evaluation methodology applied to the MedWeb Checklist dataset, the counterfactual-based test set.

Multimedia Appendix 3 Provide additional discussion about the observations from the initial audit of LLM-based annotation, and the initial audit of Qwen-based and GPT-based generated counterfactual texts. This also contains the more detailed version of the qualitative error analysis.

Multimedia Appendix 4 This documents shows the prompt templates used for data augmentation strategies. This also presents the training configurations used for fine-tuning models, and model configurations during prompt-based generation/classification. Detailed tables of the settings are included to help with reproducibility.

## Abbreviations

AF: adapter fusion
CALF: concept-aware learning framework
CDA: counterfactual data augmentation
FA: flip accuracy
Few-Shot Gen: few-shot prompt-based generative data augmentation
f-F1: F1-score for flu-or-fever detection
LLM: large language model
McouDA: multiattribute counterfactual data augmentation
MCouDA+AF: multiattribute counterfactual data augmentation with adapter fusion
m-F1: macro F1-score
MgenDA: multiattribute generative data augmentation
MGenDA+AF: multiattribute generative data augmentation with adapter fusion
MGenDA+MTL: multiattribute generative data augmentation with multitask learning training objective
MTL: fine-tuning via multitask learning framework
NLP: natural language processing
Prompt GPT: prompting GPT-4.1 for symptom classification task
Prompt Qwen: prompting Qwen2.5-7B-Instruct for symptom classification task
RoBERTa: Robustly optimized BERT approach
