# Supplementary material for: Concept-Aware Learning Framework for Detecting Common Symptoms in Social Media Text: Model Development and Evaluation Study
Source: JMIR Med Inform. 2026 Oct 15;14:e94769. doi: 10.2196/94769 (PMC13720407; doi:10.2196/94769)
Supplement: Supplementary file 1 [file medinform_v14i1e94769_app1.pdf]

Multimedia Appendix 1: Descriptive Statistics of MedWeb Data and MedWeb Checklist

Table MA1.1: Distribution of samples in the MedWeb training and test sets by symptom/disease.

	Training	Test
All	1920	640
Cold	265	90
Cough	227	80
Diarrhea	182	64
Fever	345	93
Hay Fever	163	46
Headache	251	77
Influenza	106	24
Runny Nose	375	123

Table MA1.2: Distribution of Counterfactual Sets in the Checklist Dataset by Symptom/Disease.

	No. of Sets
All	337
Cold	35
Cough	41
Diarrhea	38
Fever	50
Hay Fever	35
Headache	48
Influenza	46
Runny Nose	44
