# Supplementary material for: Concept-Aware Learning Framework for Detecting Common Symptoms in Social Media Text: Model Development and Evaluation Study
Source: JMIR Med Inform. 2026 Oct 15;14:e94769. doi: 10.2196/94769 (PMC13720407; doi:10.2196/94769)
Supplement: Supplementary file 2 [file medinform_v14i1e94769_app2.pdf]

Multimedia Appendix 2: Checklist Data Construction and Quality Assurance

The MedWeb checklist dataset was constructed by randomly sampling instances from the MedWeb test set to avoid any overlap with the training data. Sampling was performed with replacement because each selected instance functioned only as a seed text for constructing a counterfactual set. For each seed text, a target symptom or disease was randomly assigned, and all counterfactual variants in the set were generated with respect to this same target. Counterfactual texts were generated one at a time using individual and specialized prompts for each transformation. The initial versions of these texts were generated using GPT-4.1.

Each counterfactual set followed a consistent generation sequence. First, a negative example was created by removing any evidence of any symptom. Then, a target symptom is selected at random. From the negative version, a text expressing direct evidence of the target symptom was produced, followed by another expressing indirect evidence. Subsequent variants were generated by editing the direct-evidence version to reflect additional surveillance-relevant attributes, including full recovery, partial recovery, past occurrence (more than 24 hours ago), recent occurrence (within 24 hours), doubt or denial, and speculative or uncertain tone. In total, 1,000 counterfactual sets were generated following this procedure. Below is an example of a counterfactual set.

Attribute	Levels	Examples
Evidence	No Evidence	I went on a trip and got a sunburn as a souvenir.
	Direct Evidence	I went on a trip and **brought back a headache** as a souvenir.
	Indirect Evidence	**Reached for my go-to headache remedy** after that long, crowded bus ride during the trip.
Recovery	Full Recovery	I went on a trip and brought back a headache as a souvenir, **but it is completely gone now.**
	Partial Recovery	I went on a trip and brought back a headache as a souvenir; **it's still lingering but starting to get better.**
Timing	Within 24 hours	I went on a trip and developed a headache **a few hours ago**.
	Outside 24 hours	**Last weekend**, I went on a trip and brought back a headache as a souvenir.
Uncertainty	Speculative only	**If I go on a trip**, **I might** come back with a **headache** as a souvenir.
	With Doubt	I went on a trip and **maybe got a headache** as a souvenir, but I'm **not really convinced** it's anything serious.

To improve quality, we conducted three cycles of review and editing using LLM-based evaluation. Before each review phase, the researchers manually examined a small sample of texts and translated common issues into refined instructions added to the review prompt. During each review phase, the LLM rated every counterfactual text on a 7-point ordinal scale for attribute alignment, naturalness, and similarity to the seed text, and it also provided written feedback describing potential improvements. In the subsequent editing phase, the same LLM revised each text based on the feedback it had produced. This review-edit sequence was repeated three times.

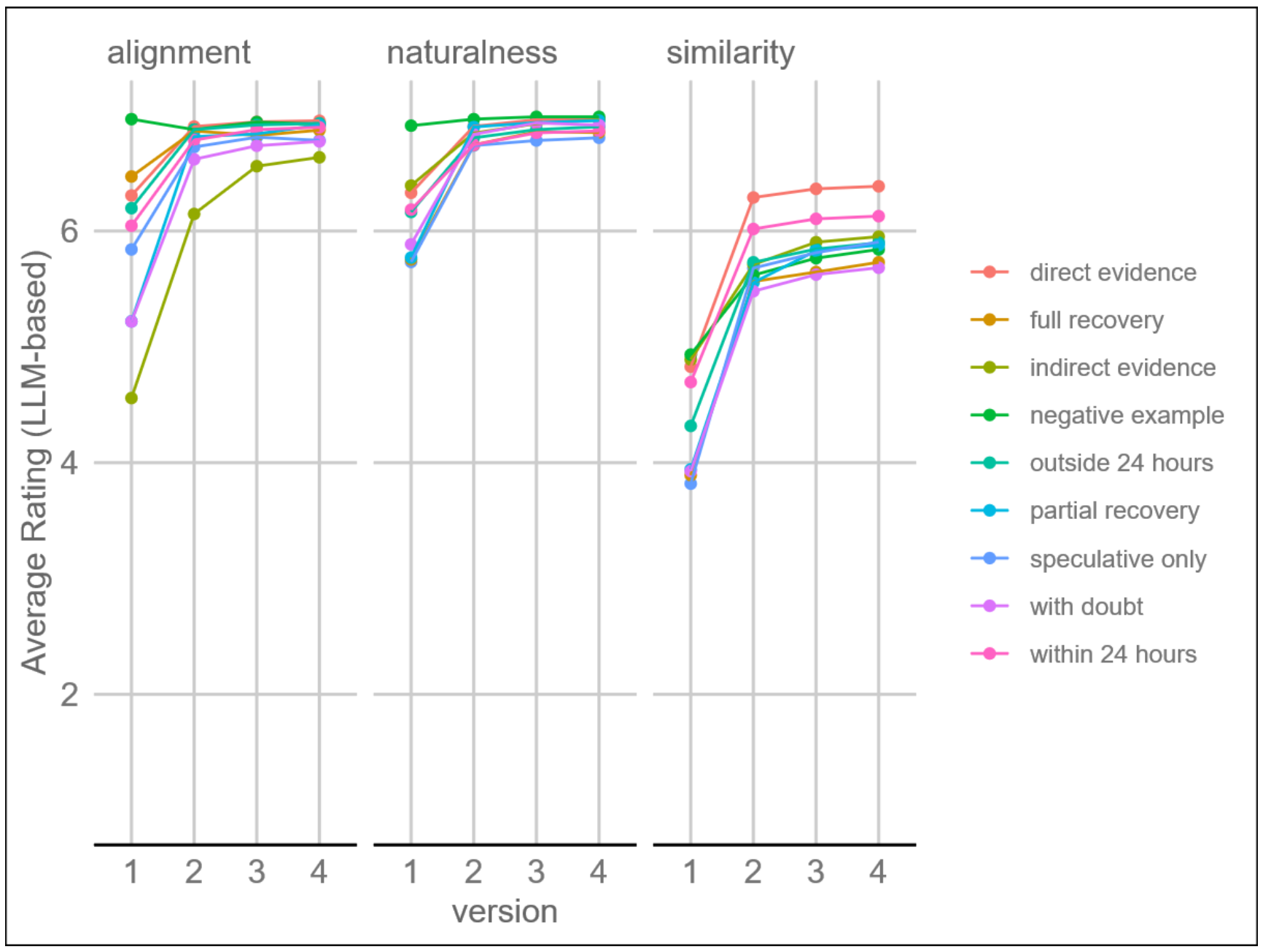

Figure MA2.1: Line plot of average ratings for each criterion (alignment, naturalness, similarity) grouped by attribute. Each group and color represents an attribute being manipulated in a single counterfactual editing task.

After the third cycle, we examined the final ratings. As shown in Figure MA2.1: Line plot of average ratings for each criterion (alignment, naturalness, similarity) grouped by attribute. Each group and color represents an attribute being manipulated in a single counterfactual editing task. In Figure MA2.1, the average scores improved across all dimensions with each iteration. To ensure dataset quality, a counterfactual set was retained only if all its texts achieved a rating of at least 5 on all dimensions. A final refinement pass was then performed using a more recent version of GPT (GPT-5), and these revised texts were considered in the final dataset.

After the final refinement pass, only counterfactual sets in which all variants received a rating of at least 5 for attribute alignment, linguistic naturalness, and semantic consistency

were retained, resulting in an initial pool of 387 sets. We then conducted an additional manual validation using 100 sampled texts. Two graduate-level researchers with NLP backgrounds independently identified the manipulated attribute in each text. Both raters identified the intended attribute in 73% of the samples for type of evidence, 98% for recovery status, 95% for timing of occurrence, and 74% for uncertainty of expression. Most disagreements involved indirect symptom expressions and uncertain statements. Nevertheless, for each attribute, at least one rater identified the intended label in more than 90% of the evaluated texts.

Finally, the researchers reviewed the rater comments and manually examined the remaining candidate sets. Texts were removed when they primarily expressed a question, described a hypothetical or pretended symptom, or referred to a nonhuman subject. Examples include “How do you say hay fever in English?”, “I pretend to have influenza,” and “I wonder if dogs get things like a stuffy nose?” This final filtering step reduced the dataset from 387 to 337 counterfactual sets.
