# Supplementary material for: Concept-Aware Learning Framework for Detecting Common Symptoms in Social Media Text: Model Development and Evaluation Study
Source: JMIR Med Inform. 2026 Oct 15;14:e94769. doi: 10.2196/94769 (PMC13720407; doi:10.2196/94769)
Supplement: Supplementary file 3 [file medinform_v14i1e94769_app3.pdf]

Multimedia Appendix 3: Manual Audit and Error Analysis

Manual Audit of LLM-based Annotation

We manually audited the GPT-4.1-generated attribute annotations for 150 posts. For each post, we reviewed the assigned labels for four surveillance-relevant attributes: type of evidence, timing of occurrence, recovery status, and epistemic stance. The audit therefore covered 600 attribute-level decisions. The objective was to assess whether the generated annotations were sufficiently reliable for use as auxiliary supervisory signals and to identify recurring sources of error.

Overall, the annotations were satisfactory for 141 of 150 posts (94.0%) for evidence, 143 posts (95.3%) for timing, 147 posts (98.0%) for recovery status, and 131 posts (87.3%) for epistemic stance. All four attribute labels were judged satisfactory for 115 posts (76.7%). As reported in the main text, recovery status was the most consistently annotated attribute, whereas epistemic stance was the most challenging. Table MA3.1 presents representative errors and borderline cases observed during the audit.

Table MA3.1: Representative errors and borderline cases in GPT-4.1-generated attribute annotations

**Error pattern**	**Example post**	**GPT-4.1 annotation**	**Audit remark**
Assertive expression classified as indirect evidence	"Finally allergies got me."	Evidence: indirect	The post directly confirms that the person is experiencing allergies, although the wording is figurative.
Symptom onset confused with current symptom presence	"I've been coughing for about 2 weeks already."	Timing: outside 24 hours	Although the cough started more than 24 hours earlier, the progressive form indicates that it is still ongoing. The symptom should therefore be treated as present within the past 24 hours.
Full recovery classified as partial recovery	"My nose isn't runny anymore, but I'm not going to work anyway."	Recovery: partially recovered	The symptom is explicitly negated. This is more appropriately labeled as fully recovered.
Uncertainty applied to the wrong proposition	"Even though I've been sick with a cold, I'm not sure if I can call in sick."	Stance: doubtful	The uncertainty concerns whether the person can miss work, not whether the person has a cold.
Question interpreted as doubt about symptom presence	"Last night I accidentally shook hands with someone who had the flu. Can you catch it from shaking hands?"	Stance: doubtful	The question concerns the mode of transmission rather than doubt about whether the referenced person had influenza.

Doubt or denial not detected	"No way, allergies? My snot is dripping like water."	Stance: certain	The opening phrase expresses disbelief or denial. A doubtful stance is more appropriate.
Generic statement interpreted as symptom evidence	"Diarrhea is the worst when you get food poisoning."	Evidence: direct	The post states a general observation and does not clearly indicate that a specific person currently has diarrhea.
Annotation triggered by a non-target condition	"I wonder if everybody had the mumps when they were little."	Evidence: indirect; timing: outside 24 hours	The attributes were assigned based on a condition outside the set of target symptoms. This suggests that the annotation should remain anchored to the surveillance targets.
Hearsay interpreted as symptom-level speculation	"They said on the news that it's easier to catch an infection during the cooler seasons. Apparently a sudden fever is a sign."	Evidence: indirect; stance: speculative	The post reports general information but does not clearly state that a person is currently experiencing fever.

Initial Audit of GPT-4 and Qwen Counterfactual Text Generation

We conducted an initial manual review of 60 multi-attribute counterfactuals: 30 generated by GPT-4.1 and 30 generated by Qwen. Each generated text was assessed based on whether it remained coherent, expressed the target symptom clearly, and preserved the original context while applying the requested changes.

GPT-4.1 produced 22 "good" counterfactuals (73.3%), whereas Qwen produced 18 "good" counterfactuals (60.0%). The most common issues involved implausible symptom substitutions, nonminimal rewriting, semantic drift, and unnatural phrasing. Representative examples are shown in Table MA3.2.

Table MA3.2. Representative issues identified during the initial audit of LLM-generated counterfactuals

**Issue**	**Generator**	**Original text**	**Generated counterfactual**	**Remark**
Implausible symptom substitution	Qwen	"Those who have to cough should wear a mask, it's good manners."	"Those who have to experience diarrhea should wear a mask, it's good manners."	The edit is minimal but the resulting context is implausible.
Nonminimal rewrite	Qwen	"My big brother gave me the flu."	"I came from a trip and got a headache this morning."	The output replaces the original context rather than editing it.

Unnatural phrasing	Qwen	“I'm not gonna worry about this endlessly runny nose.”	“I'm not gonna worry about this endlessly diarrheal condition.”	The target symptom is expressed, but the wording is unnatural.
Context mismatch	GPT-4.1	“If you can't be considerate and have manners when you cough on the train, don't even get on it.”	“If you can't be considerate and have manners when you have to take remedies for fever on the train, don't even get on it.”	The edited sentence no longer preserves the logic of the original post.
Semantic drift	GPT-4.1	“It looks like a new type of cold is spreading in Spain.”	“If I were in Spain, I might need to take some remedies for cough; hopefully, your remedy would help me recover partly.”	The informational post is changed into a hypothetical personal statement.

This preliminary review showed that both models can generate usable counterfactuals, although GPT-4.1 produced a higher proportion of satisfactory examples. The observed errors also highlight the importance of instructing the LLM to preserve the original context while applying the requested attribute changes.

Errors in the Counterfactual-based Evaluation

This section highlights the types of errors frequently observed in the baseline model and illustrates how these errors become less prominent when the model is trained on CALF. As shown in Table 9, the baseline model consistently struggles with cases requiring fine-grained distinctions. In particular, the baseline model tends to fail at cases involving recovery status, timing of occurrence, and expression of uncertainty. These weaknesses affect its ability to assign labels reliably.

The first set of examples focuses on recovery status. The baseline model often fails to differentiate between cases where the symptom is fully resolved versus cases where recovery is only partial or ongoing. The model tends to uniformly make positive predictions even when the text explicitly states that the symptom has already disappeared. In contrast, the model trained with CALF correctly distinguishes between these scenarios:

Counterfactual Pairs and Corresponding Predictions	Baseline	CALF
I went on a trip and brought back a **headache** as a souvenir, **but it's completely gone now.**	Headache [+]	Headache [-]
I went on a trip and brought back a **headache** as a souvenir; **it's still there but slowly getting better.**	Headache [+]	Headache [+]
I had a **runny nose a long time ago** and everyone in my club laughed at me, **but I've fully recovered now.**	Runny nose [+]	Runny nose [-]
I had a **runny nose** and everyone in my club laughed at me, **even though it has gotten a bit better now.**	Runny nose [+]	Runny nose [+]
I've heard of Spanish flu and Hong Kong flu. I had **influenza years ago**, but I am now **fully recovered.** Is there Japanese flu?	Influenza [-]	Influenza [-]
I've heard of Spanish flu and Hong Kong flu. I **recently had influenza** and **still feel a bit fatigued**, though I'm **mostly better now**—is there Japanese flu?	Influenza [-]	Influenza [+]

Interestingly, the baseline model is not simply "overly sensitive." In some cases, even when a recent symptom is clearly mentioned, it incorrectly predicts a negative label (see third pair in the previous table). This inconsistency suggests an unstable reliance on surface cues. The augmented MTL model, however, reliably captures the intended contrast.

A similar pattern appears in cases involving timing of occurrence, an attribute that is critical for epidemiological monitoring. As MedWeb tasks assumes daily monitoring of cases, a model that fails to distinguish between symptoms that occurred hours ago versus a week or a month ago would be suboptimal for daily surveillance tasks. The baseline model frequently misses this distinction, whereas the other model correctly identifies outdated symptoms as negative instances:

Counterfactual Pairs and Corresponding Predictions	Baseline	CALF
Allergy season is so exhausting, and I developed a **cough within the last 12 hours.**	Cough [+]	Cough [+]
**Last week**, during allergy season, I **had a persistent cough.**	Cough [+]	Cough [-]
I started experiencing **diarrhea a few hours ago.** Is it because it's cold?	Diarrhea [+]	Diarrhea [+]
A **week ago**, I **had some diarrhea**. Could it have been because it was cold?	Diarrhea [+]	Diarrhea [-]
This **cold** I came down with **this morning** is really intense, tell me if you know a good cure!	Cold [+]	Cold [+]
**Two months ago**, my **cold was** really intense, tell me if you know a good cure!	Cold [+]	Cold [-]

Finally, we examine cases that involve uncertainty of expression, where the model must distinguish pure speculation from questioning or doubtful tone only. The baseline model

tends to assign positive labels regardless of whether a symptom is mentioned only hypothetically or as a possibility. This behavior indicates insufficient sensitivity to qualifiers of uncertainty. The other model trained on counterfactually augmented data is more capable of capturing these subtle distinctions:

Counterfactual Pairs and Corresponding Predictions	Baseline	CALF
**If I go out**, I'm worried I **might** get a **fever**.	Fever [+]	Fever [-]
I'm **not even convinced** I have a **fever** right now.	Fever [+]	Fever [+]
**If my hay fever were acting up**, I might feel tired, so could you give me a treat?	Fever [+]	Fever [-]
**I probably just have hay fever**, but do I really need a treat for that?	Fever [+]	Fever [+]
**If I left Tokyo**, I might develop a **cough**.	Cough [+]	Cough [-]
I left Tokyo and **I guess** I have **a bit of a cough**, but it's **probably nothing**.	Cough [+]	Cough [+]

Overall, these examples show the possible failure points of the baseline model. Also, it shows support for the statistics observed earlier. These examples illustrate the benefit of using CALF.

Model Behavior on Real-World COVID-19 Data

Since the MedWeb Checklist relies on LLM-generated counterfactual pairs, its evaluation may not reflect the full variability of naturally occurring user-generated text. To examine whether the observed model behavior generalizes beyond synthetic perturbations, we further applied both models on the English portion of SPEED++ [49], a symptom-related event detection dataset. Although SPEED++ uses a different annotation scheme, it offers a realistic and diverse set of COVID-era posts against which we can compare how the baseline and best-performing models behave when applied to out-of-distribution symptom mentions. In this case, we focus on the differences between the cases uniquely detected by each model.

From the COVID subset of SPEED++, 69 posts were predicted positive for at least one MedWeb symptom by either model. Twenty-nine were detected by both models, ten only by the best-performing model, and thirty only by the baseline. The posts uniquely detected by the best-performing model typically contained **indirect or contextually expressed symptom evidence**, such as references to confirmed COVID infections or situations implying illness but lacking explicit symptom words. Examples include:

SPEED++ Cases and Symptom/s Detected	Baseline	CALF
"One of my closest friends passed away from **complications of the coronavirus this morning**. He had been battling cancer for a couple years…"	None	Fever
"My cousin has **tested positive for #(CoronaVirus)"**	None	Fever
"the fact i thought things would only be going better when i started taking **meds and it worked** , … but bc of the **virus situation idk** when i can and im just stuck and **starting to feel so down again**"	None	Fever
"I just **sneezed on myself.** Too risky, given the times were in. Going to wear my nose mask."	None	Runny Nose
"the fact i thought things would only be going better when i **started taking meds** and it worked , … but bc of the **virus situation** idk when i can and im just stuck and starting to **feel so down again**"	None	Fever
"My **Covid Experience** My father had **body pain and tiredness** for 2 days . I consulted local physician who **tested his temperature** and informed that **it is normal** and need not worry . My father 's **health seemed not to improve** …"	None	Fever

Interestingly, the best-performing model tends to associate "fever" with mentions of COVID infection, even though the MedWeb training data contains no COVID-related cases. This behavior is likely influenced by patterns in the training data, where several influenza-related posts imply the presence of fever without stating it explicitly. The model may have learned to infer "fever" indirectly from references to flu, and this inference appears to generalize to COVID-related contexts. However, such behavior is less likely to occur with the baseline model. On the other hand, this contextual sensitivity can also lead to errors when co-references are ambiguous. For instance, in the final example, the text describes a "COVID experience" and mentions "body pain and tiredness," but also states that the physician "tested his temperature and informed that it is normal." Predicting "fever" in this case is inconsistent with the evidence provided.

In contrast, the posts uniquely detected by the baseline model predominantly contained symptom-related vocabulary without evidence of an active or self-reported symptomatic episode. Many were informational, advisory, or educational in nature. For example, the baseline flagged the following as symptomatic:

SPEED++ Cases and Symptom/s Detected	Baseline	CALF
…There is no treatment for Covid Its Oxygen support and treatment for co morbidities Both required only if case gets serious if everybody **who had viral fever and had 103 temperature** wanted to get admitted , we whd never have any beds free	Fever	None
GET TESTED for COVID - 19 Even if you have mild symptoms **- scratchy throat , cough , runny nose or slight fever** . Mobile & amp ; pop - up testing clinics …	Cough	None

The baseline model also frequently assigned positive labels to past or fully resolved symptoms, such as: *"My family was sick in February… symptoms included headache, low fever,*

runny nose, dry cough... took me 3 weeks to get better." Additionally, the baseline tend to misclassify speculation, hypotheticals, or rhetorical questions, for example: *"bro what if i have covid - 19 and this is all just a fever dream"*. Below are other examples of these cases:

SPEED++ Cases and Symptom/s Detected	Baseline	CALF
Probably a strong possibility that I recently **had COVID - 19 a few wks ago -** I ended up w / a **dry cough** that wrecked my lungs , exhausted me for weeks , & amp ; Im still not able to take a short walk around the blk w / o nearly collapsing . This shit SUX , yet I see folks blithely ready to 1 /	Cough	None
(user) (user) **had a dry cough after returning.** This time it **lasted about 3 days.** Now I havent done the antibody test , but I would not be surprised if I did take the test and it came back (url) also now know the virus was likely here back in December .	Cough	None
can be reported as COVID - 19 with **cough as only symptom** ?(url) 3 (url)	Cough	None
Did I hear a **cough**?	Cough	None

Overall, these findings show that the baseline model is highly sensitive to the mere presence of symptom keywords, leading to false positives in posts that discuss symptoms indirectly, historically, rhetorically, or in a general advisory sense. In contrast, CALF more reliably distinguishes active symptomatic expressions from non-indicative instances such as past illness, and hypothetical statements. This behavior is desirable in surveillance settings, where misinterpreting contextual or historical mentions as active symptoms can lead to inflated estimates of apparent case counts.
