# Supplementary material for: Concept-Aware Learning Framework for Detecting Common Symptoms in Social Media Text: Model Development and Evaluation Study
Source: JMIR Med Inform. 2026 Oct 15;14:e94769. doi: 10.2196/94769 (PMC13720407; doi:10.2196/94769)
Supplement: Supplementary file 4 [file medinform_v14i1e94769_app4.pdf]

Multimedia Appendix 4: Prompts and Training Configuration

Prompt Structures

Please note that the prompt for generating a positive case is slightly different to that of a negative case. However, a similar prompt structure is applied.

Backtranslation part 1, translation to Japanese

```
 Think step by step.

    1. Your task is to translate a given English text into Japanese.

    2. Ensure that the translation is accurate and maintains the original
meaning.

    3. Ensure that it maintains the symptom or disease mentioned in the text.

    Examples:

    {examples from MedWeb Training Data}

    Given the English text, return the Japanese translation:

    English: {input text}

    Do not include any additional text or explanations, just the translation.
```

Backtranslation part 2, translation to English

```
   Think step by step.

    First, your task is to translate a given Japanese text into English.

    Examples:

    {examples from MedWeb Training Data}

    Then, paraphrase the English text while maintaining the original meaning.

    Also, ensure that it maintains the symptom or disease mentioned in the
text.

    Given the Japanese text, return the English paraphrase:

    Japanese: {Japanese translation}

    Do not include any additional text or explanations.
```

Attribute Labeling

Separate prompts were constructed for each surveillance-relevant concepts, but every prompt follows this pattern:

```
Think step by step.
1. Check if the text implies presence of {target symptom}.
2. If yes, determine whether the expression of the symptom is {attribute levels}.
It is {attribute level 1} if {definition of attribute level 1}
It is {attribute level 2} if {definition of attribute level 2}
…
{instructions about exceptions}
Examples:
Text: {example for attribute level 1}
Output: {attribute level 1}
Text: {example for attribute level 2}
Output: {attribute level 2}
Now, consider this text:
Text: {input text}
Return the appropriate label only {attribute levels}.
```

Please note that we found it more effective to use minimally contrasting examples in the prompt. For instance, below is the exact prompt for labeling direct or indirect evidence.

```
Think step by step.
1. Check if the text implies presence of {target symptom}.
2. If yes, determine whether the expression of the symptom is "direct" or "indirect".
It is "direct" if the presence of the symptom is clearly and explicitly expressed in the text
It is "indirect" if the presence of the symptom is implied only but the symptom is clearly identifiable by actions such as taking a specific medicine, or engaging in remedies.
```

If there are multiple symptoms, choose "direct" if there's at least one explicit expression of symptom.

Examples:

Text: My brother had a fever last night.

Output: direct

Text: My brother needs rest. I will buy medicines to manage his body temperature.

Output: indirect

Now, consider this text:

Text: *{input text}*

Return the appropriate label only ("direct" or "indirect").

Few-shot Generation

Think step by step.

1. Based on a sample of texts, generate a new social media post that implies the user or someone around the user has a *{target symptom}*.

2. The generated text should be in english and should not be a direct copy of the examples.

Examples of texts that imply the user has a *{target symptom}*:

{examples from MedWeb training data}

Do not include any additional text, and just generate a new text that implies the user has a *{target symptom}*.

Multi-attribute Generative Data Augmentation

Think step by step.

1. Based on a sample of texts, generate a new social media post that implies the user or someone around the user has a *{target symptom}*.

2. The generated text should be in english and should not be a direct copy of the examples.

3. *{instruction for type of evidence}*

4. The timing of the symptom is *{timing level}*.

5. The recovery status is *{recovery level}*.

6. The epistemic stance of the text is *{uncertainty level}*.

Examples of texts that imply the user has a *{target symptom}*:

{examples from MedWeb training data}

Do not include any additional text, and just generate a new text that implies the user has a *{target symptom}*.

Multi-attribute Counterfactual Data Augmentation

Think step by step.

1. Edit the given text such that a symptom is expressed with a specific set of attributes.

2. Keep edits minimal. Preserve the meaning of the original text as much as possible.

Now consider this text:

Target attributes with examples:

1. Switch the symptom from *{current symptom label}* to *{target symptom}*.

2. *{instruction for type of evidence}*

3. The timing of symptom is *{timing level}*.

Outside 24 hours: "Last month, I went to a trip and got flu."

Not specified: "I went to a trip and got flu."

Within 24 hours: "I came from a trip and got flu this morning."

4. The recovery status is *{recovery level}*.

Full recovery: "My diarrhea last night is completely healed now. Your remedy worked!"

Not specified: "My diarrhea started last night. I took some remedy."

Partial recovery: "My diarrhea last night is easing. Your remedy is helping."

5. The epistemic stance of the text is *{uncertainty level}*.

Speculative: "If I am not here tomorrow, then I may be down with a fever."

Certain: "I will not be there tomorrow. I am down with fever."

```
        Doubtful: "I may not be there tomorrow. Hopefully, this is not fever.
it can't be!"

    6. Keep the edited text as close as possible to the original text.

    Original: {input text}

    Counterfactual:

     Do not include any additional text, and just generate a counterfactual
text.
```

Training and Inference-Time Configurations

All models were fine-tuned on the research laboratory's computational server equipped with NVIDIA RTX A6000 GPUs. For the configurations used in this study, fine-tuning RoBERTa-large can be performed on a GPU with 16 GB of VRAM. Qwen2.5-7B-based synthetic data generation can also be performed on a 16 GB GPU, although quantized inference may be required depending on the input length and generation settings. In our computational environment, model training typically required approximately 30 minutes to one hour, depending on the size of the training data and the learning setup. Table MA4.1 summarizes the principal training configurations for the different fine-tuning strategies.

Table MA4.1. Summary of the principal training configurations

Setting	Standard fine-tuning	Multi-task learning	Individual adapters	AdapterFusion
Encoder parameters updated	Yes	Yes	No	No
Additional trainable components	Classification head	Main and auxiliary heads	Attribute adapter and task head	Fusion layer and symptom head
Main-task loss	BCE with logits	BCE with logits	—	BCE with logits
Auxiliary-task loss	—	Cross-entropy	Weighted cross-entropy	—
Learning rate	1×10^{-5}	1×10^{-5}	1×10^{-4}	1×10^{-5}
Batch size	16	16	16	16
Maximum sequence length	128	128	512	512
Maximum epochs	3	3	15	15
Linear scheduler with 10% warm-up	Yes	Yes	Yes	Yes
Early stopping	No	No	Yes, patience = 5	Yes, patience = 3
Seed	84	84	42	42

GPT-4.1 was accessed through the OpenAI API, whereas Qwen2.5-7B-Instruct was run locally using Hugging Face Transformers. Both models were used for synthetic-data generation and as zero-shot prompting baselines. For MGenDA, the prompt included five sampled training examples and randomly selected surveillance-relevant attribute values. For MCouDA, the prompt instead provided one seed text and instructed the model to apply a minimal counterfactual edit. The zero-shot prompting baselines used the same symptom-surveillance annotation guidelines and returned binary labels for the eight target symptoms.

Table MA4.2. Synthetic-data generation settings

Setting	GPT-4.1	Qwen2.5-7B-Instruct
Model identifier	gpt-4.1	Qwen/Qwen2.5-7B-Instruct
Inference environment	OpenAI API	Local Hugging Face inference
Synthetic-data methods	MGenDA and MCouDA	MGenDA and MCouDA
MGenDA input	Five sampled training posts with the target symptom	Five sampled training posts with the target symptom
MCouDA input	One seed post to be minimally edited	One seed post to be minimally edited
Controlled labels	Target symptom, evidence, timing, recovery status, and uncertainty	Target symptom, evidence, timing, recovery status, and uncertainty
Sampling enabled	Yes	Yes
Temperature	1.0	1.0
Top-p	1.0	1.0
Maximum generated tokens	2,048	2,048
Frequency penalty	0	Not applicable
Presence penalty	0	Not applicable
Model precision	Managed by API	Automatically selected using torch_dtype="auto"
Device mapping	Managed by API	Automatic using device_map="auto"
Random seeds used for generation batches	84 and 85	84 and 85
Generated examples per batch	1,000	1,000

Table MA4.3. Prompt-based baseline settings

Setting	GPT-4.1	Qwen2.5-7B-Instruct
Model identifier	gpt-4.1	Qwen/Qwen2.5-7B-Instruct
Inference environment	OpenAI Responses API	Local Hugging Face inference
Task	Binary symptom labeling for eight symptoms	Binary symptom labeling for eight symptoms
Prompting strategy	Zero-shot	Zero-shot
Output format	Structured output validated using a Pydantic schema	Python-style dictionary parsed and validated after generation

Decoding strategy	Deterministic	Greedy decoding
Sampling enabled	No	No
Temperature	0.0	Not applicable under greedy decoding
Beam size	Not applicable	1
Maximum output tokens	128	128
Maximum input tokens	Managed by API	1,536
Default batch size	One request per post	1
Optional quantization	Not applicable	4-bit loading supported
Retry or validation behavior	Up to 5 attempts with exponential backoff	Failed parses recorded for review
